# Aerial Oxygen-Driven Selenocyclization of *O*-Vinylanilides Mediated by Coupled Fe^3+^/Fe^2+^ and I_2_/I^−^ Redox Cycles

**DOI:** 10.3390/molecules27217386

**Published:** 2022-10-31

**Authors:** Hao-Yuan Zhang, Tong-Tong Zeng, Zhen-Biao Xie, Ying-Ying Dong, Cha Ma, Shan-Shan Gong, Qi Sun

**Affiliations:** Jiangxi Key Laboratory of Organic Chemistry, Jiangxi Science and Technology Normal University, Nanchang 330013, China

**Keywords:** diselenide, *o*-vinylanilide, redox cycle, aerial oxygen, multivalent metal, benzoxazine

## Abstract

In the past decade, selenocyclization has been extensively exploited for the preparation of a wide range of selenylated heterocycles with versatile activities. Previously, selenium electrophile-based and FeCl_3_-promoted methods were employed for the synthesis of selenylated benzoxazines. However, these methods are limited by starting material availability and low atomic economy, respectively. Inspired by the recent catalytic selenocyclization approaches based on distinctive pathways, we rationally constructed an efficient and greener double-redox catalytic system for the access to diverse selenylated benzoxazines. The coupling of I_2_/I^−^ and Fe^3+^/Fe^2+^ catalytic redox cycles enables aerial O_2_ to act as the driving force to promote the selenocyclization. Control and test redox experiments confirmed the roles of each component in the catalytic system, and a PhSeI-based pathway is proposed for the selenocyclization process.

## 1. Introduction

**Figure 1:**
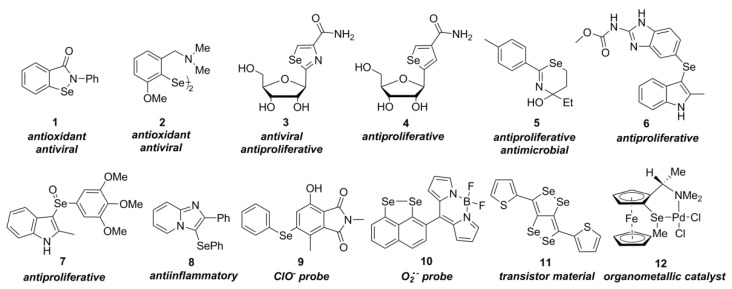
Representative organoselenium compounds.

Selenium-containing organic compounds, also known as organoselenium compounds, are an important and unique category of organic molecules. As shown in Figure 1, organoselenium compounds (**1**–**8**) exhibit versatile biological and pharmacological activities [1,2,3], such as antioxidant [4,5,6,7,8], antimicrobial [9,10,11], antiproliferative [12,13,14,15], and antiinflammatory activities [16]. Selenium-based probes (**9**–**10**) [17] have also been developed for highly sensitive fluorimetric detection of reactive oxygen species (ROS), such as ClO^−^ [18] and O_2_^•−^ [19], in living cells. In addition, the selenium-containing fused bicyclic heterocycle (**11**) and its analogs have been demonstrated as active organic field effect transistor materials [20]. In the realm of organic synthesis, organoselenium compounds also have a wide range of applications, such as ligands for organometallic catalysts (**12**) [21], synthetic intermediates [22,23,24], and even direct catalysts [25]. Therefore, a huge amount effort has been devoted to developing efficient synthetic methods for selenylated heterocycles over the past decade [26,27,28].

**Figure 2:**
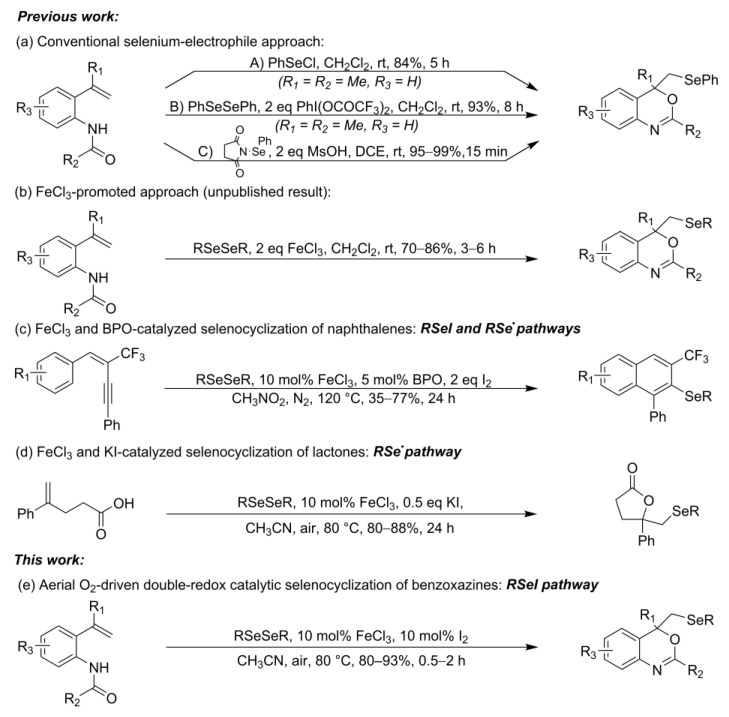
Previous and current selenocyclization methods.

As an important heterocyclic scaffold, 3,1-benzoxazine is widely found in natural products and bioactive molecules [29,30,31]. Numerous types of substituted benzoxazines have been synthesized via either cation- or radical-initiated tandem cyclizations [32,33,34,35]. Among them, selenylated benzoxazines have been successfully synthesized from the selenocyclization of selenium electrophiles, such as PhSeCl, PhSe^+^CF_3_COO^−^ [36], and *N*-(PhSe)succinimide with *o*-vinylanilides [37] (Figure 2a). However, this approach is limited by the availability of selenium electrophiles (RSeX) and difficult to apply to R-modified selenocyclizations. Fe^3+^-promoted selenocyclization with readily available diorganyl diselenides could be a good alternative approach [38,39,40,41]. In our experiments (Figure 2b), excess FeCl_3_ (2 equiv) was required to afford the desired products in 3−4 h due to the chelation of in situ-generated PhSe^−^ with Fe^3+^. Since the generation of one PhSe^+^ is accompanied by the formation of one PhSe^−^ and the consumption of one molecule of Fe^3+^, the efficiency of this method is low in terms of atomic economy. It is worth noting that Zhang and coworkers reported that the combination of a catalytic amount of FeCl_3_/benzoyl peroxide (BPO) and 2 equiv of I_2_ with diselenides afforded the desired products via both cation- and radical-initiated pathways [42] (Figure 2c). According to the mechanism proposed by Zhang et al., BPO facilitated the generation of RSeI and RSe^•^, while FeCl_3_ catalyzed the electrophilic cyclization of the neighboring aryl ring. More recently, Zhang et al. reported that I^•^ generated from the redox reaction of FeCl_3_ and KI induced the RSe^•^-initiated reaction pathway and the oxidation of the radical intermediates by Fe^3+^, and aerial oxygen yielded the desired products in 24 h under refluxing conditions [43] (Figure 2d). Inspired by these previous research, we envisioned that only catalytic amount of I_2_ is actually needed to convert diselenide to RSeI, an ideal selenium electrophile [23,44], if the resulting I^−^ could be recycled to I_2_ by a second oxidant. Many high-valent metals are capable of oxidizing I^−^, but their low-valent counterparts are prone to be oxidized by O_2_. Therefore, the insertion of a multivalent metal redox cycle into the I_2_/I^−^ redox cycle and O_2_ may construct a double-redox catalytic system for selenylated benzoxazines, featuring greener reaction conditions and high atomic economy. Herein, we report an efficient aerial O_2_-driven selenocyclization approach mediated by coupled Fe^3+^/Fe^2+^ and I_2_/I^−^ redox cycles (Figure 2e). Mechanistic investigation confirmed the roles of each component in this novel double-redox catalytic system and revealed the reasons why Fe^3+^ exhibited the best catalytic reactivity.

## 2. Results and Discussion

In the preliminary experiments, the catalytic reactivities of a series of multivalent transition metal salts, including Cu(acac)_2_, Co(acac)_3_, VO(acac)_2_, Ce(NH_4_)_2_(NO_3_)_6_, Ni(acac)_2_, MoO_2_(acac)_2_, PMA, Fe(acac)_3_, Fe(OTf)_3_, and FeCl_3_, were tested. The reaction of 1.0 equiv of *o*-vinylanilide **1a**, 0.6 equiv of diselenide **2a**, and 0.1 equiv of metal salt and I_2_ in CH_3_CN was refluxed under air atmosphere. The data summarized in Table 1 (Entry 1−10) indicate that all of these multivalent transition metals showed some catalytic effect. A major problem with these metal catalysts is that these reactions had difficulty in terms of completion. Among these metals, Fe^3+^ salts exhibited remarkably superior reactivity. In the presence of 10 mol% FeCl_3_, the selenocyclization took only 30 min to afford the desired benzoxazine product **3a** with 93% yield. In addition, the experimental results (Table 1, Entry 11–14) showed that the FeCl_3_-catalyzed reaction exhibited notable solvent effects. Acetonitrile was proved as the most suitable solvent. In addition, we further reduced the amount of FeCl_3_ and I_2_ and found that the reaction could not go to completion when the amount of either FeCl_3_ or I_2_ was lowered to 5%.

With the optimized reaction conditions, we further explored the scope of this double-redox catalytic system in the synthesis of selenylated benzoxazines. The data in Table 2 showed that the new method tolerated a variety of substituents on both *o*-vinylanilide and diorganyl diselenide substrates at different positions. Generally, both strong electron-withdrawing -NO_2_ on the benzoyl moiety and more sterically hindered phenyl on the vinyl moiety resulted in lowered reaction rate. As shown in Table 2, selenylated benzoxazines **3a**–**3t** were obtained in 80–93% yields in 0.5–2 h.

**Scheme 1:**
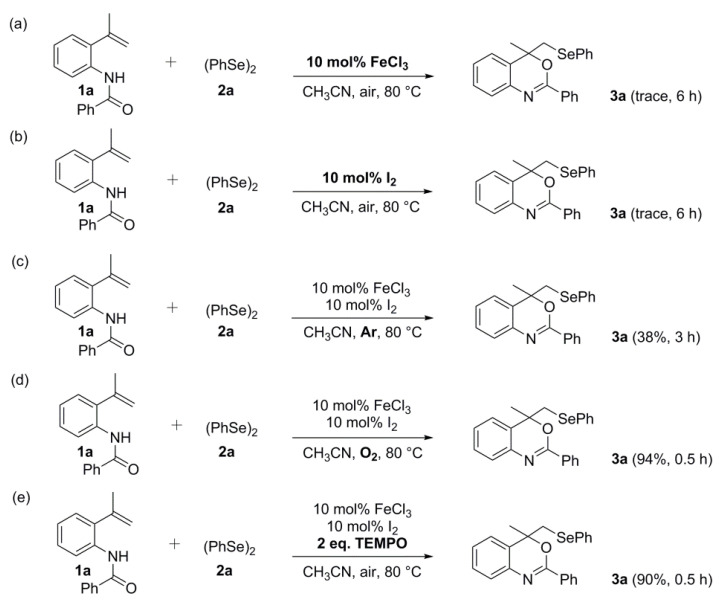
Control experiments.

To elucidate the proposed roles of each key component in the catalytic system, a series of control experiments were performed. As shown in Scheme 1a,b, the absence of either catalytic amount of FeCl_3_ or I_2_ resulted in the formation of only a trace amount of **3a**. When the reaction was conducted in argon atmosphere, it simply afforded **3a** in 38% yield (Scheme 1c). The above experimental results clearly indicate that FeCl_3_, I_2_, and aerial O_2_ are essential to the catalytic system, but replacement of aerial O_2_ with pure O_2_ did not lead to improvement in the catalytic efficacy, suggesting that the proposed O_2_ oxidation of Fe^2+^ was not the rate-limiting step (Scheme 1d). Finally, the ineffectiveness of TEMPO on the proceeding of this reaction implies that radical species (RSe^•^) should not be involved in reaction pathway [45,46] (Scheme 1e).

**Scheme 2:**
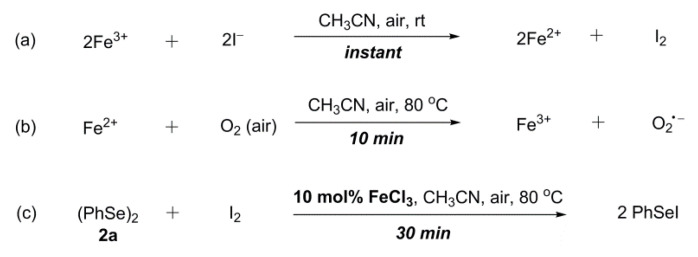
Separated control redox reactions without *o*-vinylanilide.

To prove the existence and coupling of the proposed Fe^3+^/Fe^2+^ and I_2_/I^−^ redox cycles, we further performed a series of control redox reactions without *o*-vinylanilide. As shown in Scheme 2a, a qualitative chromogenic assay (Figure S1a,b) showed that the treatment of Fe^3+^ with I^−^ at ambient temperature caused an abrupt formation of a large amount of Fe^2+^ and I_2_. In another experiment, the exposure of FeCl_2_ in CH_3_CN to air at 80 °C for 10 min led to complete oxidation of Fe^2+^ to Fe^3+^ (Scheme 2b) as indicated by a chromogenic assay (Figure S1c). Surprisingly, we found that almost no Fe^3+^ could be detected after the aqueous solution of FeCl_2_ was exposed to air for 30 min at 80 °C. This result indicates that the efficacy of the aerial oxidation of Fe^2+^ is solvent dependent, which may possibly be one important reason why CH_3_CN is most suitable for this reaction. Finally, we tested the reaction of I_2_ with PhSeSePh (Scheme 2c). As shown in Figure S2a, the reaction proceeded very slowly at ambient temperature. As indicated by the decoloration of I_2_, refluxing at 80 °C significantly promoted the formation of PhSeI. However, it still took 3 h for the reaction to reach equilibrium (Figure S2b). When a catalytic amount of Fe^3+^ (10 mol%) was added, the decoloration time was significantly shortened to 30 min (Figure S2c). This result could be explained by the specific chelation of Fe^3+^ with diselenide and induced polarization of Se-Se bonds, which have been reported in numerous previous reports [38,39,40,41,47]. The revealed dual roles of Fe^3+^ well explained its superior catalytic activity compared with other multivalent metals in this selenocyclization reaction.

**Figure 3:**
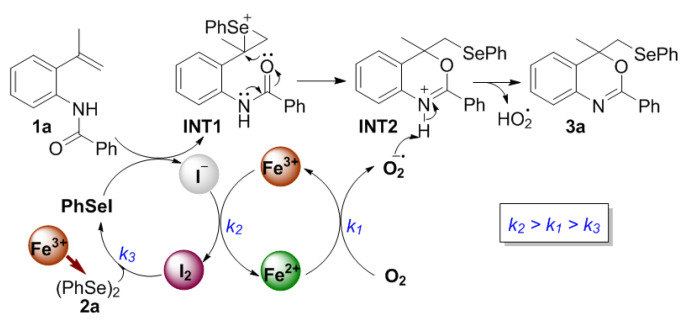
Proposed reaction mechanism.

On the basis of the above control experiments and tested redox reactions, a plausible mechanism was proposed for the current catalytic selenocyclization system (Figure 3). At the beginning of the reaction, Fe^3+^ catalyzed the formation of PhSeI (**2a**), the reactive selenium electrophile, which quickly reacted with *o*-vinylanilide (**1a**) and formed the seleniranium intermediate (**INT1**). The intramolecular nucleophilic cyclization (**INT2**) followed by the deprotonation by the superoxide radical anion (O_2_^•−^) afforded the desired benzoxazine (**3a**). Meanwhile, the released I^−^ was instantly oxidized back to I_2_ by Fe^3+^ to provide a continuous resource of PhSeI. The consumed Fe^3+^ was also quickly recycled by the aerial oxidation of Fe^2+^. Therefore, the selenocyclization was pushed forward by a green oxidant, aerial oxygen, and only catalytic amounts of FeCl_3_ and I_2_ were needed. Since *k*_2_ > *k*_1_ > *k*_3_ in the redox cycles, the majority of ion metal existed as Fe^3+^, and iodine existed as I_2_ during the reaction process, which was verified by a chromogenic assay (Figure S3).

## 3. Materials and Methods

### 3.1. General Methods

The solvents and chemical reagents used in the current research work were purchased from commercial suppliers. All of the reactions were monitored by TLC plates coated with 0.25 mm silica gel 60 F_254_ and visualized by 254 nm UV. The silica gel used in column chromatography (particle size 32–63 μm) was purchased from Qingdao Haiyang Chemicals, China. ^1^H, ^13^C, and ^19^F NMR spectra were recorded on an AV-400 instrument (Bruker BioSpin, Faellanden, Switzerland) with chemical shifts referenced to DMSO-*d_6_* or CDCl_3_ and reported in parts per million. Infrared spectra were obtained with a Vertex-70 instrument (Bruker Optics, Billerica, MA, US). HRMS spectra were acquired with a micrOTOF-Q II instrument (Bruker Daltonics, Billerica, MA, US)and reported as *m*/*z*. Melting points were measured on anX-4 melting point apparatus and were uncorrected (Tech Instrument, Beijing, China).The characterization data of new *o*-vinylanilides [37,48,49,50,51] including **1e**, **1f**, **1g**, and **1h** and known selenylated benzoxazines [36,52] including **3a**, **3d**, **3q**, and **3r** are listed in the Supplementary Materials. The NMR spectra of new *o*-vinylanilides and benzoxazines are provided in the Supplementary Materials.

### 3.2. General Synthetic Procedure and the Characterization of Selenylated Benzoxazines (***3a***–***3t***)

To a mixture of *o*-vinylanilide (0.1 mmol) and diorganyl diselenide (0.06 mmol) in CH_3_CN (2 mL), FeCl_3_ (0.01 mmol) and I_2_ (0.01 mmol) were added. The reaction was heated at 80 °C for 0.5–2 h and then concentrated *in vacuo*. Flash column chromatography on silica gel (PE/EA = 10:1) afforded the desired products **3a**–**3t** in pure form.

4-Methyl-2-(4-nitrophenyl)-4-((phenylselanyl)methyl)-4*H*-benzo[*d*][1,3]oxazine (**3b**): a yellow oil. ^1^H NMR (400 MHz, CDCl_3_): *δ* 8.23 (s, 4H), 7.39–7.35 (m, 4H), 7.28–7.22 (m, 1H), 7.17–7.12 (m, 4H), 3.58 (d, *J* = 13.0 Hz, 1H), 3.38 (d, *J* = 13.0 Hz, 1H), 1.92 (s, 3H); ^13^C NMR (100 MHz, CDCl_3_): *δ* 153.9, 149.2, 147.0, 138.4, 138.2, 132.8 (×2), 129.2, 129.0, 128.7 (×3), 127.6, 127.1 (×2), 125.8, 124.4, 123.9, 123.2 (×2), 119.1, 80.8, 59.5, 29.6; IR (KBr): *v*_max_ 3464, 3088, 3002, 2935, 1635, 1590, 1522, 1385, 1346, 1185, 1085, 939, 893, 810, 745 cm^−1^; HRMS (ESI+): *m*/*z* calcd for C_22_H_19_N_2_O_3_Se [M + H]^+^ 439.0555; found 439.0552.

4-Methyl-4-((phenylselanyl)methyl)-2-(thiophen-2-yl)-4*H*-benzo[*d*][1,3]oxazine (**3c**): a colorless oil. ^1^H NMR (400 MHz, CDCl_3_): *δ* 7.60 (d, *J* = 2.2 Hz, 1H), 7.38 (d, *J* = 3.9 Hz, 1H), 7.31–7.30 (m, 2H), 7.21–7.18 (m, 2H), 7.06 (t, *J* = 4.8 Hz, 5H), 6.99 (t, *J* = 3.7 Hz, 1H), 3.49 (d, *J* = 10.2 Hz, 1H), 3.26 (d, *J* = 10.2 Hz, 1H), 1.79 (s, 3H); ^13^C NMR (100 MHz, CDCl_3_): *δ* 151.9, 137.7, 135.8, 131.8 (×2), 129.4, 129.2, 129.0, 128.0, 127.9 (×3), 127.6, 126.6, 125.9, 125.3, 123.9, 122.1, 79.4, 38.6, 25.3; IR (KBr): *v*_max_ 3724, 2962, 1589, 1479, 1426, 1367, 1314, 1263, 1218, 1028, 804 cm^−1^; HRMS (ESI+): *m*/*z* calcd for C_22_H_18_NOSSe [M + H]^+^ 400.0269; found 400.0266.

8-Methyl-6-phenyl-8-((phenylselanyl)methyl)-8*H*-[1,3]dioxolo[4′,5′:4,5]benzo[1,2-*d*][1,3]oxazine (**3e**): a yellow oil. ^1^H NMR (400 MHz, CDCl_3_): *δ* 8.10 (d, *J* = 7.5 Hz, 2H), 7.48–7.38 (m, 5H), 7.15–7.14 (m, 3H), 6.82 (s, 1H), 6.61 (s, 1H), 5.95 (d, *J* = 12.8 Hz, 1H), 5.92 (d, *J* = 12.8 Hz, 1H), 3.48 (d, *J* = 12.8 Hz, 1H), 3.35 (d, *J* = 12.8 Hz, 1H), 1.84 (s, 1H); ^13^C NMR (100 MHz, CDCl_3_): *δ* 155.0, 147.7, 146.1, 134.0, 132.8 (×2), 132.5, 131.1, 130.4, 128.9 (×3), 128.1 (×2), 127.8 (×2), 126.9, 121.7, 106.2, 103.5, 101.3, 79.9, 39.5, 26.4; IR (KBr): *v*_max_ 3722, 2963, 1624, 1593, 1482, 1401, 1317, 1262, 1162, 1091, 1019, 805, 765 cm^−1^; HRMS (ESI+): *m*/*z* calcd for C_23_H_20_NO_3_Se [M + H]^+^ 438.0603; found 438.0604.

6-(4-Chlorophenyl)-8-methyl-8-((phenylselanyl)methyl)-8*H*-[1,3]dioxolo[4′,5′:4,5]benzo[1,2-*d*][1,3]oxazine (**3f**): a yellow oil. ^1^H NMR (400 MHz, CDCl_3_): *δ* 7.99 (d, *J* = 8.5 Hz, 2H), 7.38–7.35 (m, 4H), 7.16–7.13 (m, 3H), 6.79 (s, 1H), 6.59 (s, 1H), 5.95 (d, *J* = 1.2 Hz, 1H), 5.93 (d, *J* = 1.2 Hz, 1H), 3.46 (d, *J* = 12.9 Hz, 1H), 3.31 (d, *J* = 12.9 Hz, 1H), 1.83 (s, 1H); ^13^C NMR (100 MHz, CDCl_3_): *δ* 154.0, 147.7, 146.2, 137.2, 133.8, 132.7 (×2), 131.0, 130.3, 129.1 (×2), 129.0 (×3), 128.4 (×2), 127.0, 121.6, 106.2, 103.4, 101.3, 80.1, 39.6, 26.5; IR (KBr): *v*_max_ 3721, 2891, 1574, 1478, 1441, 1368, 1312, 1263, 1192, 1036, 939, 863, 830, 736 cm^−1^; HRMS (ESI+): *m*/*z* calcd for C_23_H_21_ClNO_3_Se [M + H]^+^ 474.8455; found 474.8457.

8-Methyl-8-((phenylselanyl)methyl)-6-(thiophen-2-yl)-8*H*-[1,3]dioxolo[4′,5′:4,5]benzo [1,2-*d*][1,3]oxazine (**3g**): a yellow oil. ^1^H NMR (400 MHz, CDCl_3_): *δ* 7.64 (d, *J* = 3.4 Hz, 1H), 7.45 (d, *J* = 4.8 Hz, 1H), 7.40–7.37 (m, 2H), 7.16–7.15 (m, 3H), 7.06 (t, *J* = 4.2 Hz, 1H), 6.76 (s, 1H), 6.59 (s, 1H), 5.94 (s, 1H), 5.92 (s, 1H), 3.47 (d, *J* = 12.8 Hz, 1H), 3.32 (d, *J* = 12.8 Hz, 1H), 1.82 (s, 1H); ^13^C NMR (100 MHz, CDCl_3_): *δ* 151.7, 147.7, 145.9, 136.9, 133.9, 132.7 (×2), 130.4, 129.9, 129.7, 128.9 (×3), 127.6, 126.9, 121.6, 105.9, 103.6, 101.2, 80.4, 39.3, 26.3; IR (KBr): *v*_max_ 3725, 2944, 1626, 1591, 1478, 1421, 1365, 1308, 1263, 1183, 1035, 852, 804, 734 cm^−1^; HRMS (ESI+): *m*/*z* calcd for C_21_H_18_NO_3_SSe [M + H]^+^ 444.0167; found 444.0163.

6-(4-Methoxyphenyl)-8-methyl-8-((phenylselanyl)methyl)-8*H*-[1,3]dioxolo[4′,5′:4,5]benzo [1,2-*d*][1,3]oxazine (**3h**): a yellow oil. ^1^H NMR (400 MHz, CDCl_3_): *δ* 8.05 (d, *J* = 8.7 Hz, 2H), 7.41–7.39 (m, 2H), 7.17–7.16 (m, 3H), 6.93 (d, *J* = 8.7 Hz, 2H), 6.80 (s, 1H), 6.62 (s, 1H), 5.96 (s, 1H), 5.94 (s, 1H), 3.88 (s, 3H), 3.48 (d, *J* = 12.7 Hz, 1H), 3.34 (d, *J* = 12.7 Hz, 1H), 1.84 (s, 3H); ^13^C NMR (100 MHz, CDCl_3_): *δ* 162.1, 155.0, 147.7, 145.7, 134.3, 132.7 (×2), 129.6 (×2), 128.9 (×3), 126.9, 113.5 (×2), 105.9, 103.4, 101.2, 79.7, 55.3, 39.4, 26.2; IR (KBr): *v*_max_ 3715, 2963, 1601, 1505, 1478, 1367, 1310, 1258, 1172, 1088, 1032, 941, 763 cm^−1^; HRMS (ESI+): *m*/*z* calcd for C_24_H_22_NO_4_Se [M + H]^+^ 468.0709; found 468.0712.

2,4-Diphenyl-4-((phenylselanyl)methyl)-4*H*-benzo[*d*][1,3]oxazine (**3i**): a colorless oil. ^1^H NMR (400 MHz, CDCl_3_): *δ* 8.17 (d, *J* = 8.3 Hz, 2H), 7.49 (d, *J* = 7.0 Hz, 1H), 7.45–7.37 (m, 8H), 7.32–7.26 (m, 3H), 7.22–7.14 (m, 5H), 3.95 (d, *J* = 13.1 Hz, 1H), 3.90 (d, *J* = 13.1 Hz, 1H); ^13^C NMR (100 MHz, CDCl_3_): *δ* 156.2, 142.3, 139.4, 133.4 (×3), 132.3, 131.4 (×2), 130.4, 129.2, 129.0 (×2), 128.4 (×2), 128.2 (×2), 128.0 (×2), 127.5, 127.1, 126.4, 126.0, 125.5, 124.6, 83.5, 39.3; IR (KBr): *v*_max_ 3515, 3438, 1956, 1628, 1482, 1455, 1316, 1260, 1082, 1022, 801, 763 cm^−1^; HRMS (ESI+): *m*/*z* calcd for C_27_H_22_NOSe [M + H]^+^ 456.0861; found 456.0862.

4-Phenyl-4-((phenylselanyl)methyl)-2-(*p*-tolyl)-4*H*-benzo[*d*][1,3]oxazine (**3j**): a colorless oil. ^1^H NMR (400 MHz, CDCl_3_): *δ* 8.14 (d, *J* = 8.2 Hz, 2H), 7.52–7.48 (m, 1H), 7.43 (t, *J* = 7.5 Hz, 2H), 7.34–7.26 (m, 4H), 7.19–7.18 (m, 2H), 7.09–7.05 (m, 1H), 7.02–6.97 (m, 1H), 3.60 (d, *J* = 12.6 Hz, 1H), 3.43 (d, *J* = 12.6 Hz, 1H), 1.94 (s, 3H); ^13^C NMR (100 MHz, CDCl_3_): *δ* 156.1, 138.8, 135.8, 132.5, 132.3 (×2), 131.3, 130.7, 129.4, 129.1 (×2), 128.6 (×3), 128.1 (×2), 128.0 (×2), 127.7, 127.0, 126.6, 125.3, 123.0, 79.8, 38.2, 26.6; IR (KBr): *v*_max_ 3436, 1628, 1484, 1446, 1385, 1318, 1262, 1162, 1089, 1024, 758 cm^−1^; HRMS (ESI+): *m*/*z* calcd for C_28_H_24_NOSe [M + H]^+^ 470.1018; found 470.1015.

2-(4-Chlorophenyl)-4-phenyl-4-((phenylselanyl)methyl)-4*H*-benzo[*d*][1,3]oxazine (**3k**): a colorless oil. ^1^H NMR (400 MHz, CDCl_3_): *δ* 8.07 (d, *J* = 8.6 Hz, 2H), 7.44–7.36 (m, 8H), 7.33–7.29 (m, 3H), 7.24–7.12 (m, 5H), 3.93 (d, *J* = 13.2 Hz, 1H), 3.89 (d, *J* = 13.2 Hz, 1H); ^13^C NMR (100 MHz, CDCl_3_): *δ* 155.2, 142.1, 139.2, 137.6, 133.3 (×2), 130.8, 129.3 (×2), 129.0 (×2), 128.5 (×3), 128.4 (×2), 128.3 (×2), 127.4, 127.2, 126.6, 125.9, 125.6, 124.6, 83.7, 39.1; IR (KBr): *v*_max_ 3399, 1658, 1481, 1446, 1398, 1315, 1259, 1085, 1022, 939, 801, 736 cm^−1^; HRMS (ESI+): *m*/*z* calcd for C_27_H_21_ClNOSe [M + H]^+^ 490.0471; found 490.0472.

2-(4-Nitrophenyl)-4-phenyl-4-((phenylselanyl)methyl)-4*H*-benzo[*d*][1,3]oxazine (**3l**): a colorless oil. ^1^H NMR (400 MHz, CDCl_3_): *δ* 8.24 (s, 4H), 7.44–7.27 (m, 10H), 7.20–7.13 (m, 4H), 3.94 (s, 2H); ^13^C NMR (100 MHz, CDCl_3_): *δ* 154.0, 149.4, 142.0, 138.8, 133.2 (×2), 130.6, 129.4, 129.0 (×2), 128.7 (×2), 128.5 (×3), 127.4, 127.2, 126.0, 125.9 (×2), 124.8, 123.9, 123.3 (×2), 84.3, 39.0; IR (KBr): *v*_max_ 3399, 2963, 1628, 1521, 1499, 1446, 1390, 1316, 1260, 1083, 1024, 801, 762 cm^−1^; HRMS (ESI+): *m*/*z* calcd for C_27_H_21_N_2_O_3_Se [M + H]^+^ 501.0712; found 501.0709.

4-Phenyl-4-((phenylselanyl)methyl)-2-(thiophen-2-yl)-4*H*-benzo[*d*][1,3]oxazine (**3m**): a colorless oil. ^1^H NMR (400 MHz, CDCl_3_): *δ* 7.75 (d, *J* = 3.6 Hz, 1H), 7.48–7.45 (m, 3H), 7.41–7.26 (m, 8H), 7.23–7.17 (m, 4H), 7.15–7.09 (m, 2H), 3.95 (d, *J* = 13.1 Hz, 1H), 3.86 (d, *J* = 13.1 Hz, 1H); ^13^C NMR (100 MHz, CDCl_3_): *δ* 152.9, 142.1, 139.5, 133.3 (×2), 130.9, 130.4, 130.2, 129.3, 129.0 (×2), 128.4 (×3), 128.3 (×2), 127.7, 127.3, 127.1, 126.2, 126.0, 125.3, 124.7, 83.9, 39.1; IR (KBr): *v*_max_ 3401, 2963, 1628, 1521, 1465, 1400, 1261, 1088, 1025, 802, 755 cm^−1^; HRMS (ESI+): *m*/*z* calcd for C_25_H_20_NOSSe [M + H]^+^ 462.0425; found 462.0415.

6-Bromo-2,4-diphenyl-4-((phenylselanyl)methyl)-4*H*-benzo[*d*][1,3]oxazine (**3n**): a colorless oil. ^1^H NMR (400 MHz, CDCl_3_): *δ* 8.06 (d, *J* = 6.2 Hz, 2H), 7.43 (t, *J* = 6.0 Hz, 1H), 7.38–7.32 (m, 5H), 7.29 (d, *J* = 5.8 Hz, 2H), 7.25–7.20 (m, 3H),7.15–7.07 (m, 5H), 3.81 (d, *J* = 10.6 Hz, 1H), 3.77 (d, *J* = 10.6 Hz, 1H); ^13^C NMR (100 MHz, CDCl_3_): *δ* 155.4, 140.5, 137.5, 132.6 (×2), 131.2, 130.8, 130.6 (×2), 129.4, 128.2, 128.0 (×3), 127.5 (×2), 127.4 (×2), 127.2 (×2), 127.0, 126.6, 126.3, 126.1, 124.9, 118.2, 82.3, 38.1; IR (KBr): *v*_max_ 3758, 2921, 1617, 1573, 1471, 1385, 1315, 1257, 1176, 1156, 1079, 1024, 966, 827, 735 cm^−1^; HRMS (ESI+): *m*/*z* calcd for C_27_H_21_BrNOSe [M + H]^+^ 533.9966; found 533.9949.

6-Bromo-2-methyl-4-phenyl-4-((phenylselanyl)methyl)-4*H*-benzo[*d*][1,3]oxazine (**3o**): a colorless oil. ^1^H NMR (400 MHz, CDCl_3_): *δ* 7.36–7.35 (m, 2H), 7.30–7.23 (m, 6H), 7.15–7.14 (m, 3H), 6.94 (d, *J* = 6.2 Hz, 2H), 3.68 (d, *J* = 10.6 Hz, 1H), 3.63 (d, *J* = 10.6 Hz, 1H), 1.97 (s, 3H); ^13^C NMR (100 MHz, CDCl_3_): *δ* 159.0, 141.0, 136.8, 132.4 (×2), 131.2, 129.4 (×2), 128.0 (×2), 127.6, 127.5 (×2), 127.3, 126.8, 126.4, 125.2, 124.9, 117.9, 82.0, 38.0, 20.6; IR (KBr): *v*_max_ 3722, 2890, 1598, 1574, 1478, 1411, 1368, 1312, 1263, 1193, 1036, 936, 863, 831, 783 cm^−1^; HRMS (ESI+): *m*/*z* calcd for C_22_H_19_BrNOSe [M + H]^+^ 471.9810; found 471.9803.

4-(((2-Chlorophenyl)selanyl)methyl)-4-methyl-2-phenyl-4*H*-benzo[*d*][1,3]oxazine (**3p**): a colorless oil. ^1^H NMR (400 MHz, CDCl_3_): *δ* 8.14 (d, *J* = 7.7 Hz, 3H), 7.50–7.42 (m, 3H), 7.34–7.27 (m, 4H), 7.19 (s, 2H), 7.07 (t, *J* = 7.4 Hz, 1H), 6.99 (t, *J* = 7.6 Hz, 1H), 3.60 (d, *J* = 12.6 Hz, 1H), 3.42 (d, *J* = 12.6 Hz, 1H), 1.94 (s, 3H); ^13^C NMR (100 MHz, CDCl_3_): *δ* 156.1, 147.0, 138.8, 132.4, 131.2, 129.4, 128.1 (×2), 128.0 (×2), 127.7, 126.6, 125.3, 124.4, 123.9, 123.0, 119.1, 79.8, 38.2, 26.6; IR (KBr): *v*_max_ 3742, 2942, 2883, 1624, 1593, 1572, 1487, 1451, 1319, 1284, 1245, 1173, 1092, 1067, 1029, 935, 819, 760 cm^−1^; HRMS (ESI+): *m*/*z* calcd for C_22_H_19_ClNOSe [M + H]^+^ 428.0315; found 428.0307.

6-(4-Methoxyphenyl)-8-methyl-8-((methylselanyl)methyl)-8*H*-[1,3]dioxolo[4′,5′:4,5]benzo[1,2-*d*][1,3]oxazine (**3s**): a yellow oil. ^1^H NMR (400 MHz, CDCl_3_): *δ* 8.10 (d, *J* = 8.9 Hz, 2H), 6.95 (d, *J* = 8.8 Hz, 2H), 6.82 (s, 1H), 6.69 (s, 1H), 5.98 (s, 2H), 3.97 (s, 3H), 3.05 (d, *J* = 13.0 Hz, 1H), 2.95 (d, *J* = 13.0 Hz, 1H), 1.84 (d, *J* = 8.7 Hz, 6H); ^13^C NMR (100 MHz, CDCl_3_): *δ* 162.2, 155.1, 147.6, 145.8, 134.4, 129.6 (×2), 121.9, 113.6 (×2), 105.9, 103.4, 101.2, 80.0, 55.3, 36.9, 31.4, 30.2, 26.0, 6.4; IR (KBr): *v*_max_ 3741, 3410, 2960, 2899, 2836, 1614, 1505, 1479, 1311, 1256, 1172, 1086, 1034, 941, 838, 806 cm^−1^; HRMS (ESI+): *m*/*z* calcd for C_19_H_20_NO_4_Se [M + H]^+^ 406.0552; found 406.0550.

4-(((4-Methoxyphenyl)selanyl)methyl)-4-methyl-2-phenyl-4*H*-benzo[*d*][1,3]oxazine (**3t**): a yellow oil. ^1^H NMR (400 MHz, CDCl_3_): *δ* 8.30 (d, *J* = 7.2 Hz, 2H), 7.55–7.48 (m, 3H), 7.44 (d, *J* = 8.8 Hz, 2H), 7.39 (d, *J* = 4.1 Hz, 2H), 7.28–7.23 (m, 1H), 7.19 (d, *J* = 7.4 Hz, 1H), 6.84 (d, *J* = 8.6 Hz, 2H), 3.80 (s, 3H), 3.69 (d, *J* = 11.0 Hz, 1H), 3.50 (d, *J* = 11.0 Hz, 1H), 1.96 (s, 3H); ^13^C NMR (100 MHz, CDCl_3_): *δ*159.2, 156.0, 138.8, 134.6, 132.3 (×2), 131.6, 129.5, 128.4 (×2), 128.2 (×2), 127.0, 126.8, 125.6, 123.0, 122.1 115.0 (×2), 55.3, 26.6, 15.7; IR (KBr): *v*_max_ 3727, 3046, 2996, 2941, 2907, 2837, 1932, 1849, 1794, 1618, 1592, 1272, 1489, 1448, 1369, 1318, 1029, 967, 838, 890, 835, 814, 751 cm^−1^; HRMS (ESI+): *m*/*z* calcd for C_23_H_22_NO_2_Se [M + H]^+^ 424.0810; found 424.0805.

## 4. Conclusions

In summary, a novel double-redox catalytic system was rationally constructed to provide efficient access to a variety of selenylated benzoxazines. The combination of only catalytic amounts of FeCl_3_ and I_2_ and the use of aerial oxygen as the end oxidant make this approach greener and more atomically efficient than conventional methods based on selenium electrophiles and FeCl_3_. This new method is widely applicable to a great diversity of *o*-vinylanilide and diorganyl diselenide substrates. Mechanistic investigation confirmed that the coupling of I_2_/I^−^ and Fe^3+^/Fe^2+^ catalytic redox cycles enabled aerial O_2_ to act as the driving force to promote the selenocyclization reaction, which proceeds via a PhSeI-based pathway.

## Figures and Tables

**Table 1:** Optimization of reaction conditions ^1^.

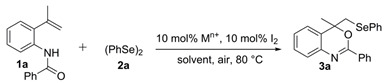				
Entry	M^n+^	Solvent	Reaction Time (h)	Isolated Yield (%)
1	Cu(acac)_2_	CH_3_CN	8	55
2	Co(acac)_3_	CH_3_CN	8	51
3	VO(acac)_2_	CH_3_CN	12	45
4	Ce(NH_4_)_2_(NO_3_)_6_	CH_3_CN	2	52
5	Ni(acac)_2_	CH_3_CN	8	22
6	MoO_2_(acac)_2_	CH_3_CN	8	27
7	PMA	CH_3_CN	5	28
8	Fe(OTf)_3_	CH_3_CN	9	92
9	Fe(acac)_3_	CH_3_CN	1	94
10	FeCl_3_	CH_3_CN	0.5	93
11	FeCl_3_	DCE	3	55
12	FeCl_3_	THF	4	73
13	FeCl_3_	DMSO	3	80
14	FeCl_3_	EtOH	6	81

^1^ Reaction conditions: **1a** (0.10 mmol), **2a** (0.06 mmol), FeCl_3_ (0.01 mmol), I_2_ (0.01 mmol), solvent (2.0 mL), 80 °C, air.

**Table 2:** Aerial O_2_-driven double-redox synthesis of selenylated benzoxazines (**3a**–**3t**).

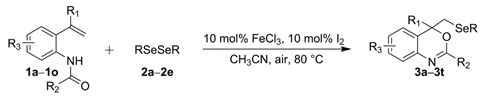
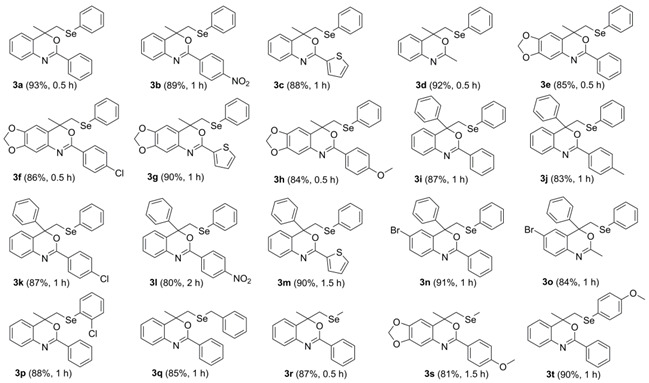

## Data Availability

Not applicable.

## Supplementary Materials

The following are available online at https://www.mdpi.com/article/10.3390/molecules27217386/s1: characterization data of new *o*-vinylanilides and known selenylated benzoxazines, Figures S1–S3: The chromogenic assays of the control redox reactions without *o*-vinylanilide and Figures S4–S51: The NMR spectra of new *o*-vinylanilides and all products.

## Sample Availability

Samples of all compounds are available from the authors.
