# Supplementary material for: Aerial Oxygen-Driven Selenocyclization of O-Vinylanilides Mediated by Coupled Fe3+/Fe2+ and I2/I− Redox Cycles
Source: Molecules. 2022 Oct 31;27(21):7386. doi: 10.3390/molecules27217386 (PMC9656128; doi:10.3390/molecules27217386)
Supplement: Supplementary file 1 [file molecules-27-07386-s001.zip › molecules-1995601-supplementary.pdf]

Supporting Information

Aerial Oxygen-Driven Selenocyclization of *o*-Vinylanilides Mediated by Coupled Fe^{3+}/Fe^{2+} and I_2/I^- Redox Cycles

Hao-Yuan Zhang, Tong-Tong Zeng, Zhen-Biao Xie, Ying-Ying Dong, Cha Ma, Shan-Shan Gong and Qi Sun *

Jiangxi Key Laboratory of Organic Chemistry, Jiangxi Science and Technology Normal University, Nanchang 330013, China
* Correspondence: sunqi@jxstnu.edu.cn

Table of contents

1. General synthetic procedures and characterization of *o*-vinylanilides — Page S2–S3
2. Characterization of known selenylated benzoxazines — Page S3–S4
3. Qualitative chromogenic assays for control redox reactions without *o*-vinylanilide (Figure S1–S3) — Page S5–S6
4. ^{1}H and ^{13}C spectra of new *o*-vinylanilides (Figure S4–S11) — Page S7–S9
5. ^{1}H and ^{13}C spectra of selenylated benzoxazines (Figure S12–S51) — Page S10–S29
6. References — Page S30

1. General synthetic procedures and characterization of *o*-vinylanilides

1.1 General synthetic procedures[1]

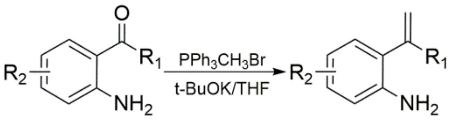

Step 1: To a mixture of 2-aminoacetophenones (22.2 mmol), methyltriphenyl-phosphonium bromide (33.3 mmol) was added potassium *t*-butylate (1.0 M in THF, 34 mL) under argon atmosphere. The reaction was stirred overnight at ambient temperature. The reaction solution was concentrated *in vacuo* and redissolved in ethyl acetate (20 mL). The ethyl acetate solution was washed with saturated aqueous $NaHCO_3$ (20 mL). The organic phase was dried over anhydrous $MgSO_4$ and concentrated *in vacuo*. Flash column chromatography afforded desired *o*-aminostyrene intermediates.

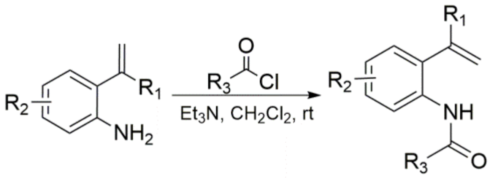

Step 2: To a solution of 2-aminostyrenes (18.9 mmol) and Et_3N (28.3 mmol) in CH_2Cl_2 (60 mL) was added benzoylchloride (22.7 mmol) at 0 °C. The reaction was stirred for 3 h at ambient temperature. The reaction solution was concentrated *in vacuo*. Flash column chromatography afforded *o*-vinylanilides (**1a–1o**) in pure form.

The characterization data of known *o*-vinylanilides (**1a–1d** and **1i–1o**) are in agreement with previous reports[2–5].

1.2 Characterization of new *o*-vinylanilides (1e–1h)

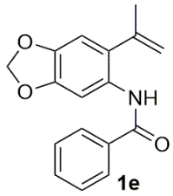

1e

***N*-(6-(Prop-1-en-2-yl)benzo[*d*][1,3]dioxol-5-yl)benzamide (1e)**: a white solid; mp 77–80 °C; ^{1}H NMR (400 MHz, $CDCl_3$): δ 8.36 (br, 1H), 8.04 (s,1H), 7.82 (t, J = 7.4 Hz, 2H), 7.57–7.47 (m, 3H), 6.67 (s, 1H), 5.97 (s, 2H), 5.45 (s, 1H), 5.09 (s, 1H), 2.08(s, 3H); ^{13}C NMR (100 MHz, $CDCl_3$) δ 164.8, 146.8, 143.9, 143.2, 135.0, 131.7, 128.8, 128.2, 127.1, 126.8, 116.9, 107.4, 103.1, 101.3, 24.5; IR (KBr): υ_{max} 3717, 2663, 1948, 1529, 1307, 1244, 1188, 1131, 1040, 904, 846 cm^{-1}; HRMS (ESI+): *m*/*z* calcd for $C_{17}H_{16}NO_3$ [M+H]$^+$ 282.1125; found 282.1128.

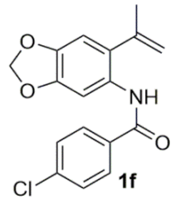

1f

4-Chloro-*N*-(6-(prop-1-en-2-yl)benzo[*d*][1,3]dioxol-5-yl)benzamide (1f): a white solid;

mp 83–85 °C; ^{1}H NMR (400 MHz, $CDCl_3$): *δ* 8.30 (br, 1H), 8.01 (s, 1H), 7.76 (d, *J* = 8.4 Hz, 2H), 7.47 (d, *J* = 8.4 Hz, 2H), 6.68 (s, 1H), 5.99 (s, 1H), 5.46 (s, 2H), 5.08 (s, 1H), 2.08 (s, 3H); ^{13}C NMR (100 MHz, $CDCl_3$): *δ* 163.8, 146.9, 144.1, 143.3, 138.0, 133.4, 129.1, 128.3, 127.9, 127.2, 116.9, 107.4, 103.1, 101.3, 24.5; IR (KBr): v_{max} 3716, 2919, 1532, 1529, 1360, 1261, 1191, 1131, 1037, 904, 846 cm^{-1}; HRMS (ESI+): *m*/*z* calcd for $C_{17}H_{15}ClNO_3$ $[M+H]^+$ 316.0735; found 316.0731.

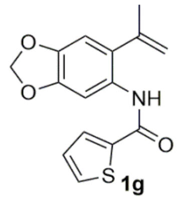

1g

***N*-(6-(Prop-1-en-2-yl)benzo[*d*][1,3]dioxol-5-yl)thiophene-2-carboxamide (1g)**: a white solid; mp 91–93 °C; ^{1}H NMR (400 MHz, $CDCl_3$): *δ* 8.22 (br, 1H), 7.98 (s, 1H), 7.54–7.50 (m, 2H), 7.13 (t, *J* = 4.0 Hz, 1H), 6.67 (s, 1H), 5.98 (s, 2H), 5.47 (s, 1H), 5.09 (s, 1H), 2.09 (s, 3H); ^{13}C NMR (100 MHz, $CDCl_3$): *δ* 159.3, 146.8, 144.0, 143.2, 139.6, 130.4, 128.1, 127.8, 127.0, 116.9, 115.4, 107.3, 103.1, 101.3, 100.5, 97.8, 24.5; IR (KBr): v_{max} 3678, 2895, 1654, 1505, 1424, 1357, 1240, 1182, 1132, 1038, 860, 728 cm^{-1}; HRMS (ESI+): *m*/*z* calcd for $C_{15}H_{14}NO_3S$ $[M+H]^+$ 288.0689; found 288.0690.

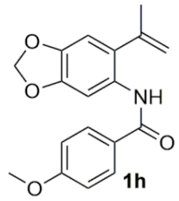

1h

4-Methoxy-*N*-(6-(prop-1-en-2-yl)benzo[*d*][1,3]dioxol-5-yl)benzamide (1h): a white solid; mp 75–77 °C; ^{1}H NMR (400 MHz, $CDCl_3$): *δ* 8.26 (bs, 1H), 8.03 (s, 1H), 7.79 (t, *J* = 8.7 Hz, 2H), 6.99 (t, *J* = 8.7 Hz, 2H), 6.67 (d, 1H), 5.98 (s, 1H), 5.45 (s, 1H), 5.09 (s, 1H), 3.89 (s, 3H), 2.08 (s, 3H); ^{13}C NMR (100 MHz, $CDCl_3$): *δ* 164.4, 162.4, 146.8, 143.8, 143.4, 132.8, 128.7, 127.0, 116.8, 114.0, 107.3, 103.1, 101.2, 55.4, 24.5; IR (KBr): v_{max} 3716, 2862, 1650, 1594, 1505, 1483, 1426, 1360, 1292, 1239, 1182, 1094, 1038, 934, 861 cm^{-1}; HRMS (ESI+): *m*/*z* calcd for $C_{18}H_{18}NO_4$ $[M+H]^+$ 312.1230; found 312.1231.

2. Characterization of known selenylated benzoxazines[6,7]

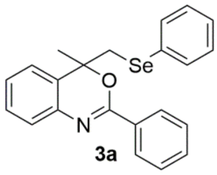

3a

4-Methyl-2-phenyl-4-((phenylselanyl)methyl)-4*H*-benzo[*d*][1,3]oxazine (3a): a colorless oil. ^{1}H NMR (400 MHz, $CDCl_3$): *δ* 8.01 (d, *J* = 8.0 Hz, 2H), 7.53 (t, *J* = 8.0 Hz, 1H), 7.45 (t, *J* = 8.0 Hz, 2H), 7.42–7.40 (m, 2H), 7.36 (d, *J* = 8.0 Hz, 1H), 7.34–7.30 (m, 1H), 7.24–7.20 (m, 2H), 7.19–7.15 (m, 3H), 3.74 (d, J = 12.0 Hz, 1H), 3.57 (d, *J* = 12.0 Hz, 1H), 1.80 (s, 3H); ^{13}C NMR (100 MHz, $CDCl_3$): *δ* 155.3, 138.9, 131.1, 130.7, 132.6, 131.5, 128.2, 129.0, 128.8, 128.5,

127.4, 127.2, 126.8, 124.9, 123.9, 80.3, 27.3; IR (KBr): υ_{max} 3422, 3064, 3031, 2951, 1692, 1588, 1570, 1523, 1455, 1400, 1340, 1252, 1177, 1136, 1092, 1043, 1013, 989, 899, 832, 755 cm^{-1}; HRMS (ESI+): *m*/*z* calcd for $C_{22}H_{20}NOSe$ $[M+H]^+$ 394.0706; found 394.0710.

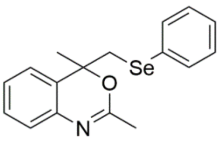

3d

2,4-Dimethyl-4-phenylselenomethyl-4*H*-benzo[*d*][1,3]oxazine (3d): a colorless oil. ^1H NMR (400 MHz, $CDCl_3$): *δ* 7.42–7.40 (m, 2H), 7.26–7.20 (m, 4H), 7.11 (d, *J* = 7.4 Hz, 2H), 7.03 (d, *J* = 7.0 Hz, 1H), 3.46 (d, *J* = 12.9 Hz, 1H), 3.24 (d, *J* = 12.9 Hz, 1H), 1.92 (s, 3H), 1.78 (s, 3H); ^{13}C NMR (100 MHz, $CDCl_3$): *δ* 159.8, 138.4, 133.4, 130.6, 129.2, 129.1, 128.1, 127.2, 126.4, 124.5, 123.2, 79.9, 40.6, 27.2, 21.6; IR (KBr): υ_{max} 3422, 3065, 3031, 2949, 1692, 1587, 1570, 1528, 1455, 1411, 1345, 1255, 1180, 1135, 1092, 1047, 1020, 999, 879, 852, 753 cm^{-1}; HRMS(ESI+): *m*/*z* calcd for $C_{17}H_{18}NOSe$ $[M+H]^+$ 331.0475; found 331.0477.

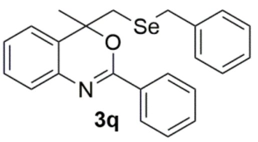

3q

4-((Benzylselanyl)methyl)-4-methyl-2-phenyl-4*H*-benzo[*d*][1,3]oxazine (3q): a colorless oil. ^1H NMR (400 MHz, $CDCl_3$): *δ* 8.13 (d, *J* = 8.0 Hz, 2H), 7.57–7.50 (m, 3H), 7.35–7.23 (m, 6H), 7.14 (t, *J* = Hz, 3H), 3.63–3.57 (m, 2H), 3.19 (d, *J* = 12.0 Hz, 1H), 3.04 (d, *J* = 12.0 Hz, 1H), 1.76 (s, 3H); ^{13}C NMR (100 MHz, $CDCl_3$): *δ* 155.4, 139.3, 138.5, 132.3, 131.8, 129.2, 129.0, 128.9, 128.6, 128.5, 127.7, 126.9, 126.7, 124.8, 123.9, 80.5, 35.0, 27.9, 26.8; IR (KBr): υ_{max} 3750, 2956, 2853, 1645, 1578, 1545, 1445, 1445, 1315, 125, 1245, 1178, 1045, 1087, 1082, 955, 829, 770 cm^{-1}; HRMS (ESI+): *m*/*z* calcd for $C_{23}H_{22}NOSe$ $[M+H]^+$ 408.0862; found 408.0869.

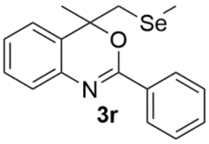

3r

4-Methyl-4-((methylselanyl)methyl)-2-phenyl-4*H*-benzo[*d*][1,3]oxazine (3r): a colorless oil. ^1H NMR (400 MHz, $CDCl_3$): *δ* 8.11 (d, *J* = 8.0 Hz, 2H), 7.57–7.54 (m, 1H), 7.50 (d, *J* = 8.0 Hz, 2H), 7.31 (d, *J* = 8.0 Hz, 2H), 7.24–7.20 (m, 2H), 3.22 (d, *J* = 16.0 Hz, 1H), 3.08 (d, *J* = 16.0 Hz, 1H), 1.80–1.77 (m, 6H); ^{13}C NMR (100 MHz, $CDCl_3$): *δ* 155.3, 138.4, 132.3, 131.7, 129.2, 128.9, 128.6, 127.6 (×2), 126.8, 124.7, 123.9, 80.7, 36.8, 26.9, 5.9; IR (KBr): υ_{max} 3750, 2956, 2853, 1645, 1578, 1545, 1445, 1445, 1315, 125, 1245, 1178, 1045, 1087, 1082, 955, 829, 770 cm^{-1}; HRMS (ESI+): *m*/*z* calcd for $C_{17}H_{18}NOSe$ $[M+H]^+$ 332.0549; found 332.0550.

3. Qualitative chromogenic assays for control redox reactions without *o*-vinylanilide

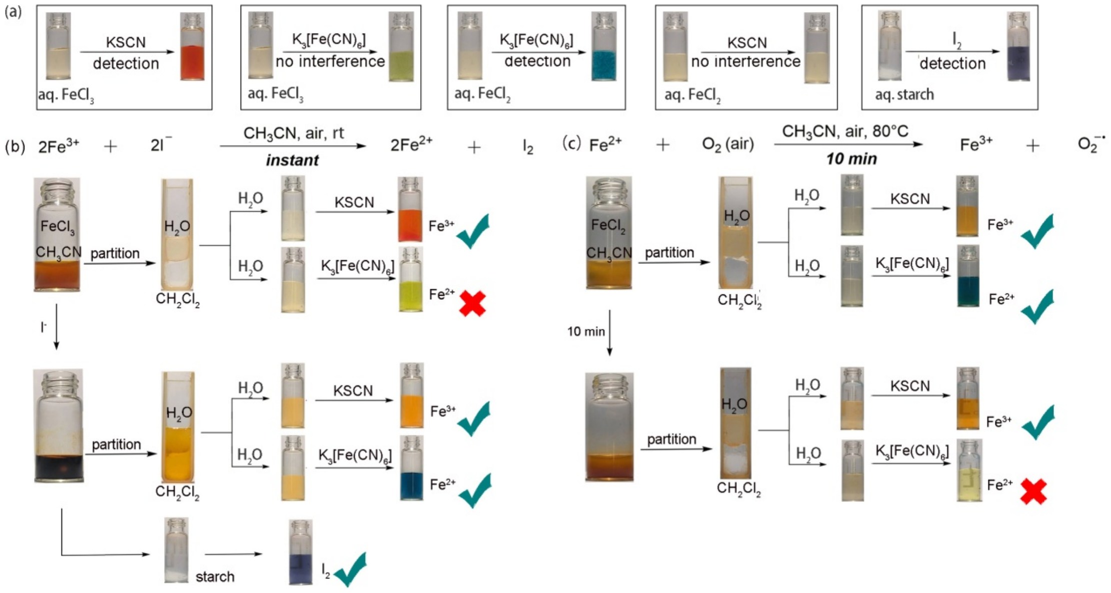

Figure S1. (a) General methods for chromogenic detection of Fe^{3+}, Fe^{2+}, and I_2[a,b]; (b) Qualitative chromogenic assay for the reaction of Fe^{3+} and I^- in CH_3CN; (c) qualitative chromogenic assay for the reaction of Fe^{2+} and aerial O_2 in CH_3CN. [a]Conditions for qualitative detection of Fe^{3+} and Fe^{2+}: [Fe^{3+}] and [Fe^{2+}]=0.005 M, [KSCN]=[K_3FeCN_6]=0.1 M, and KSCN=3 eq./K_3FeCN_6=1.5 eq. [b]Conditions for qualitative detection of I_2: [I_2]= 0.005 M ; [starch]= 10 mg/mL.

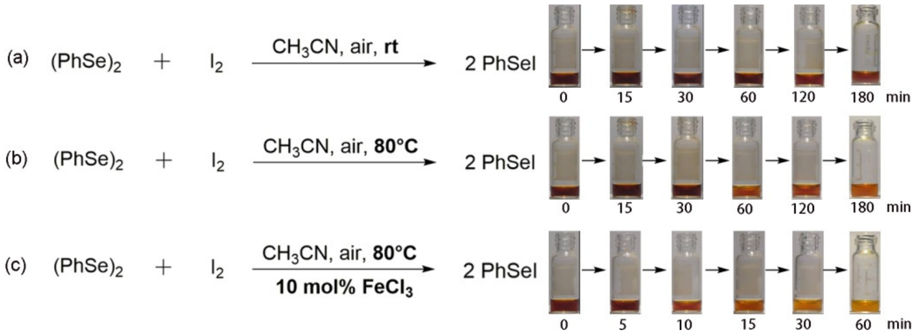

Figure S2. The reaction of I_2 with PhSeSePh at ambient temperature (a), 80 °C (b), and 80 °C with 10 mol% $FeCl_3$ (c).

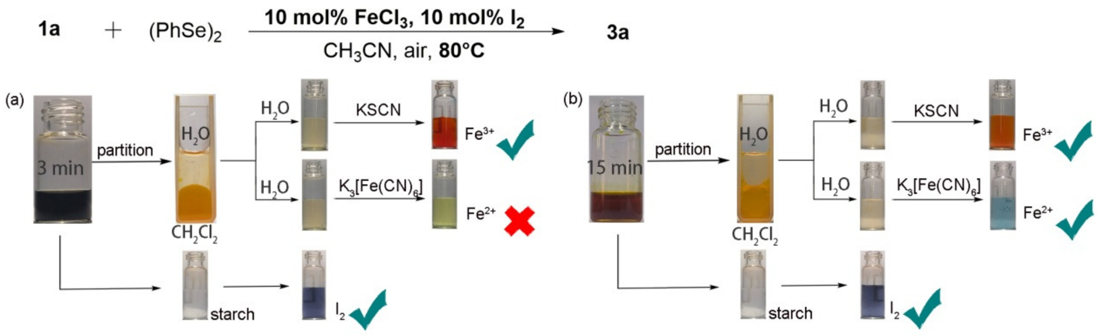

Figure S3. The chromogenic assay for the monitoring the changes of key redox species at 3 min (a) and 15 min (b) during the reaction process.

4. ^{1}H and ^{13}C spectra of new *o*-vinylanilides

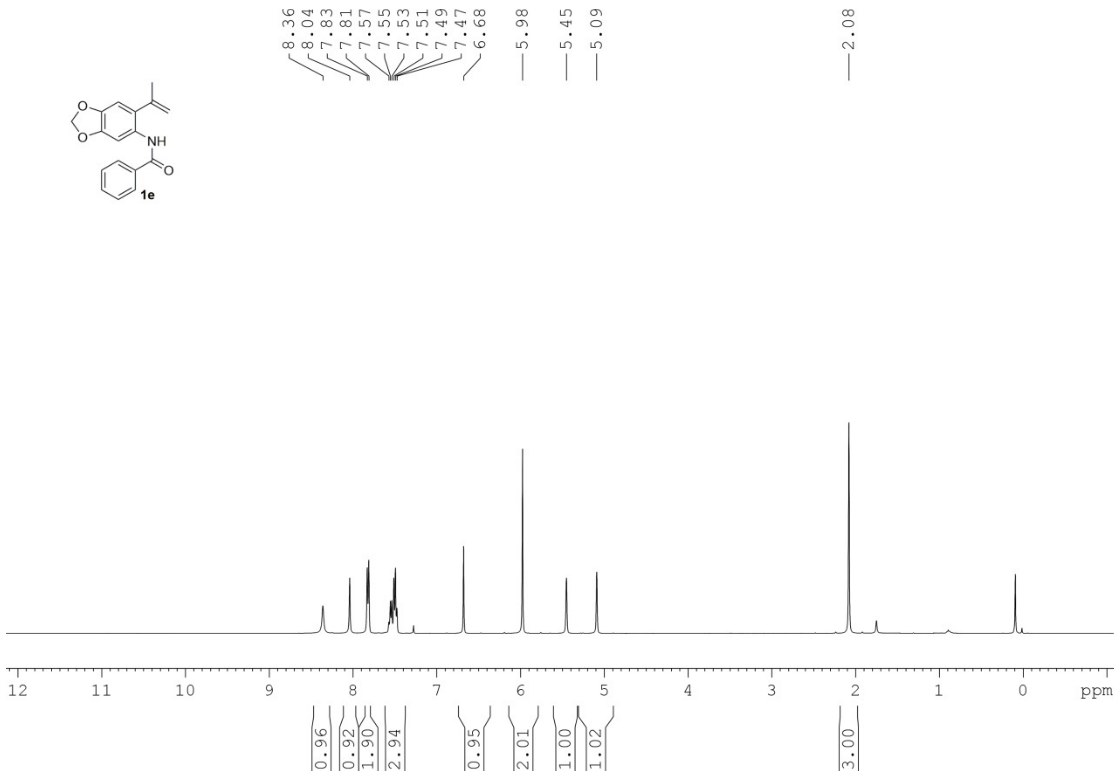

Figure S4. ^{1}H NMR spectrum of **1e**

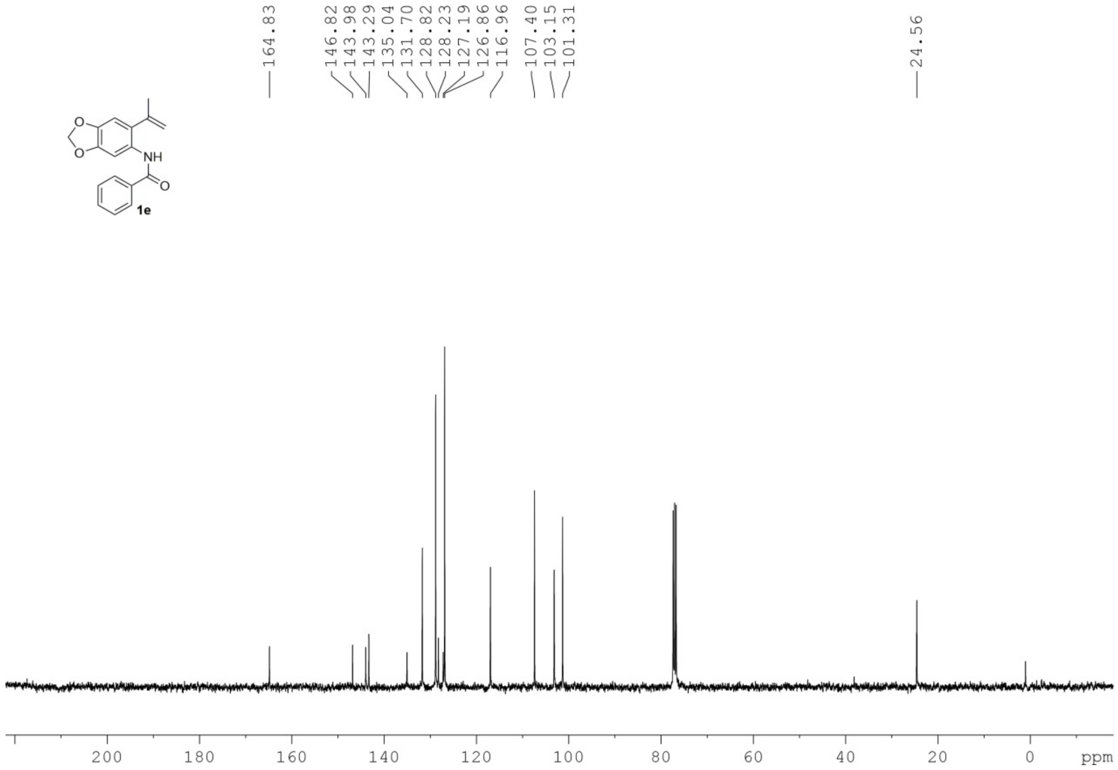

Figure S5. ^{13}C NMR spectrum of **1e**

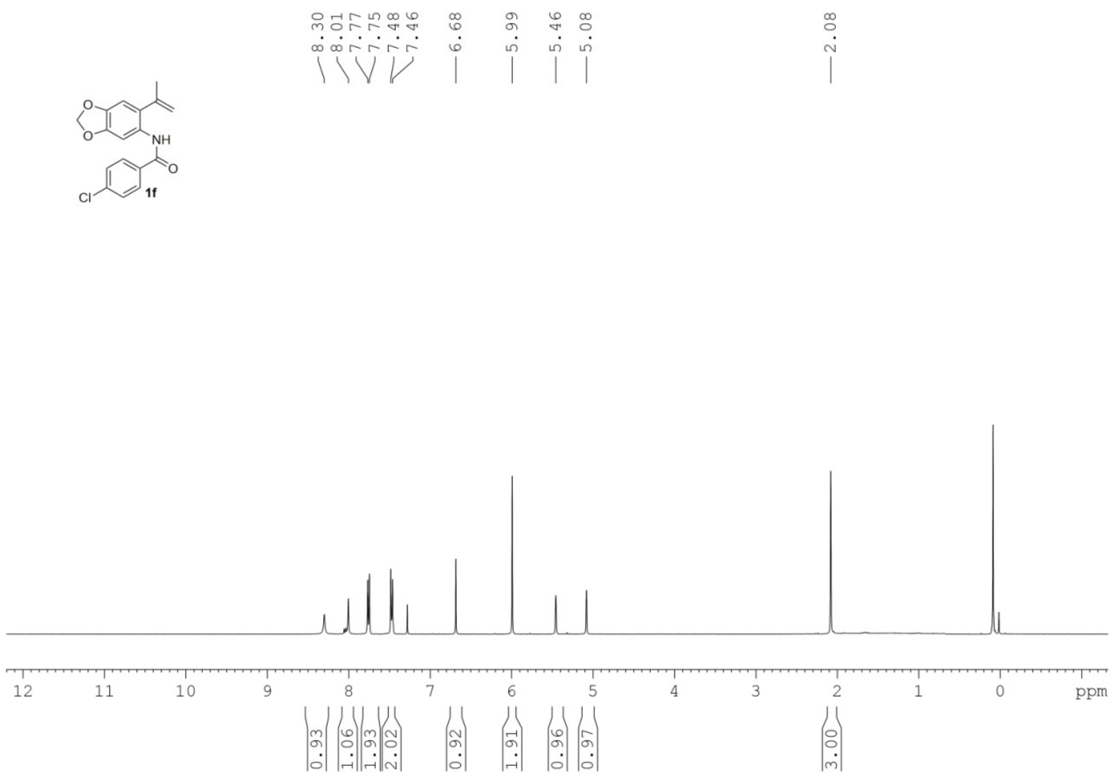

Figure S6. ^{1}H NMR spectrum of **1f**

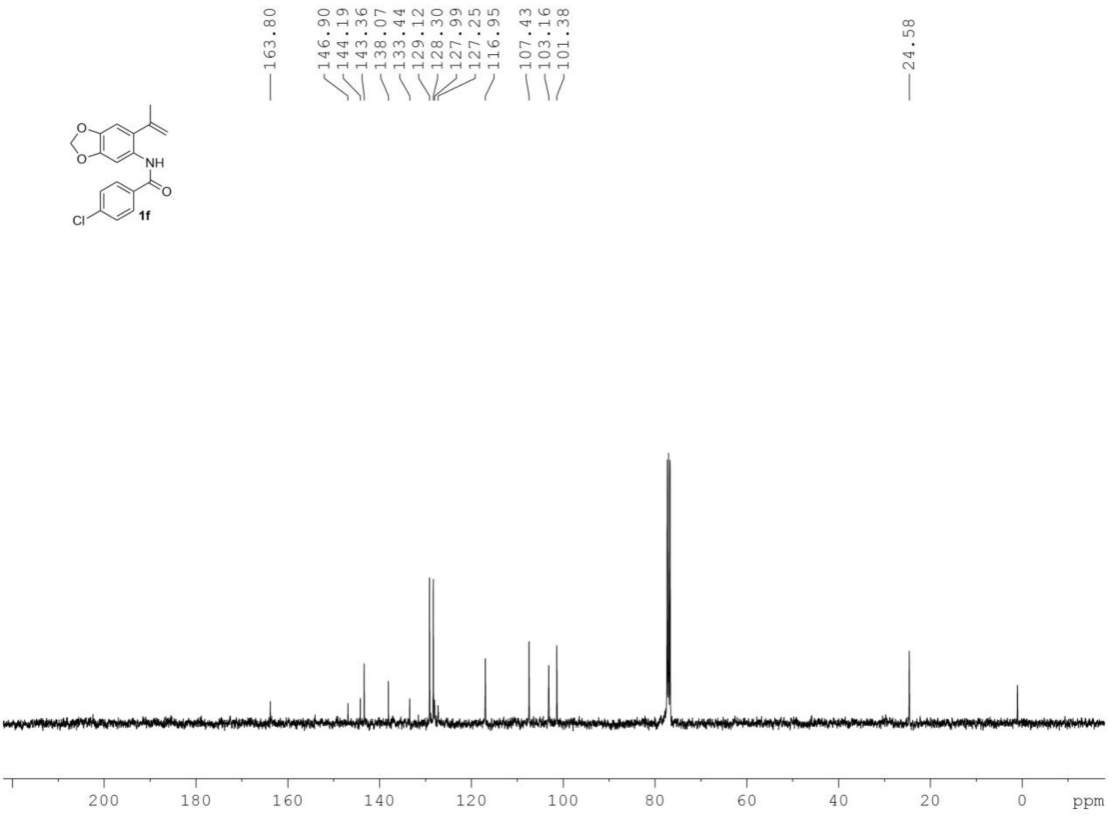

Figure S7. ^{13}C NMR spectrum of **1f**

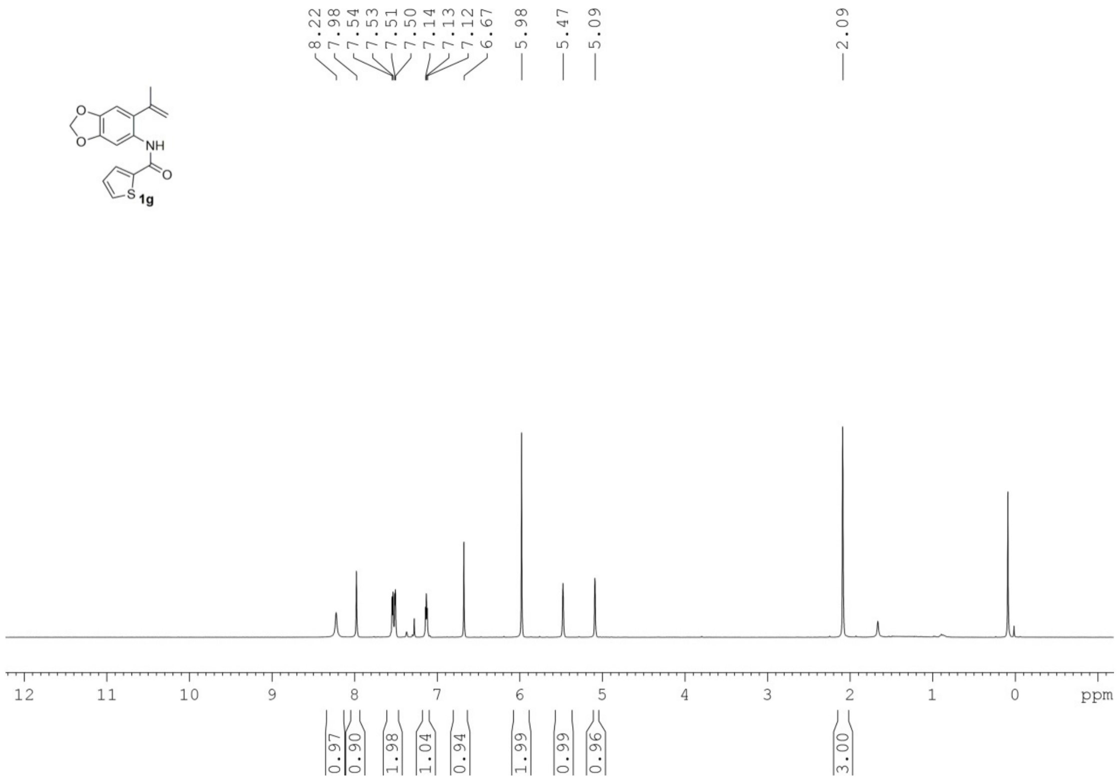

Figure S8. ^{1}H NMR spectrum of **1g**

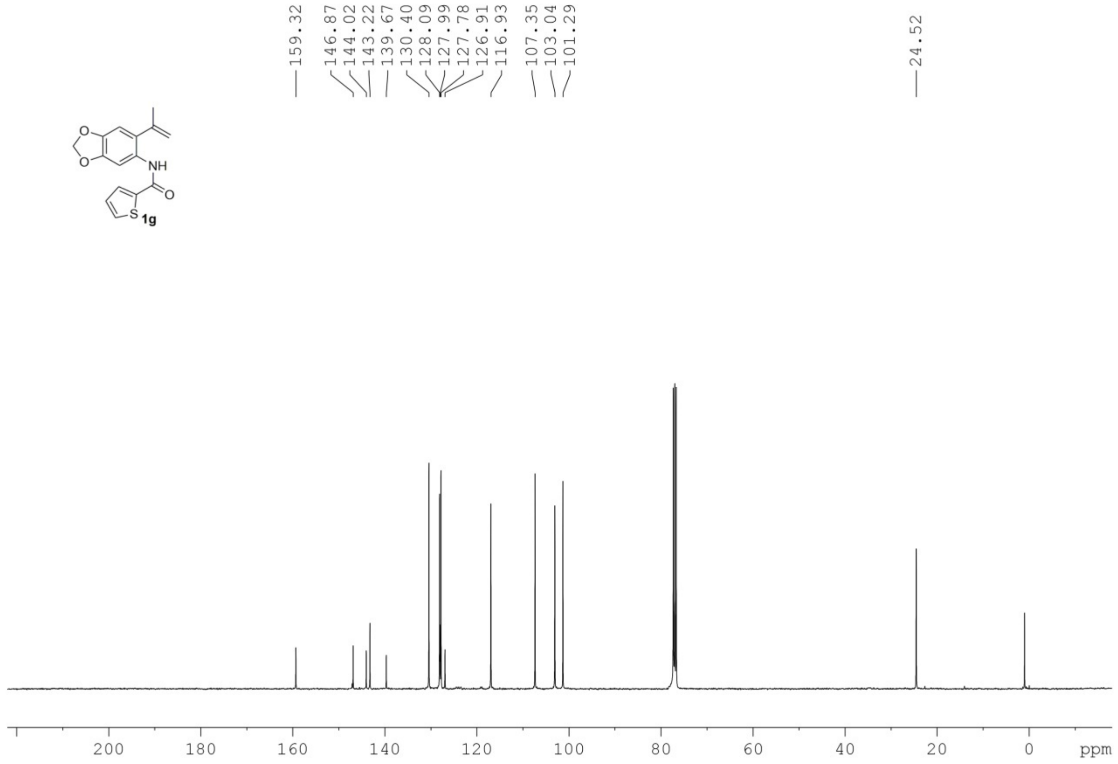

Figure S9. ^{13}C NMR spectrum of **1g**

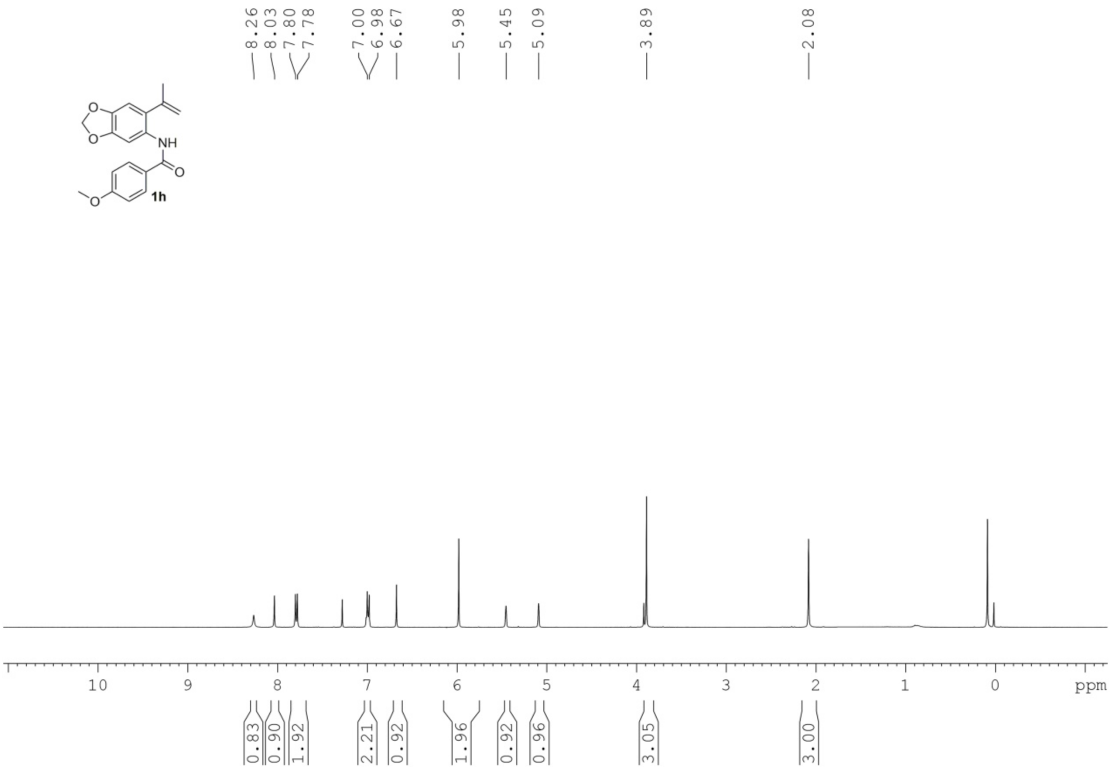

Figure S10. ^{1}H NMR spectrum of **1h**

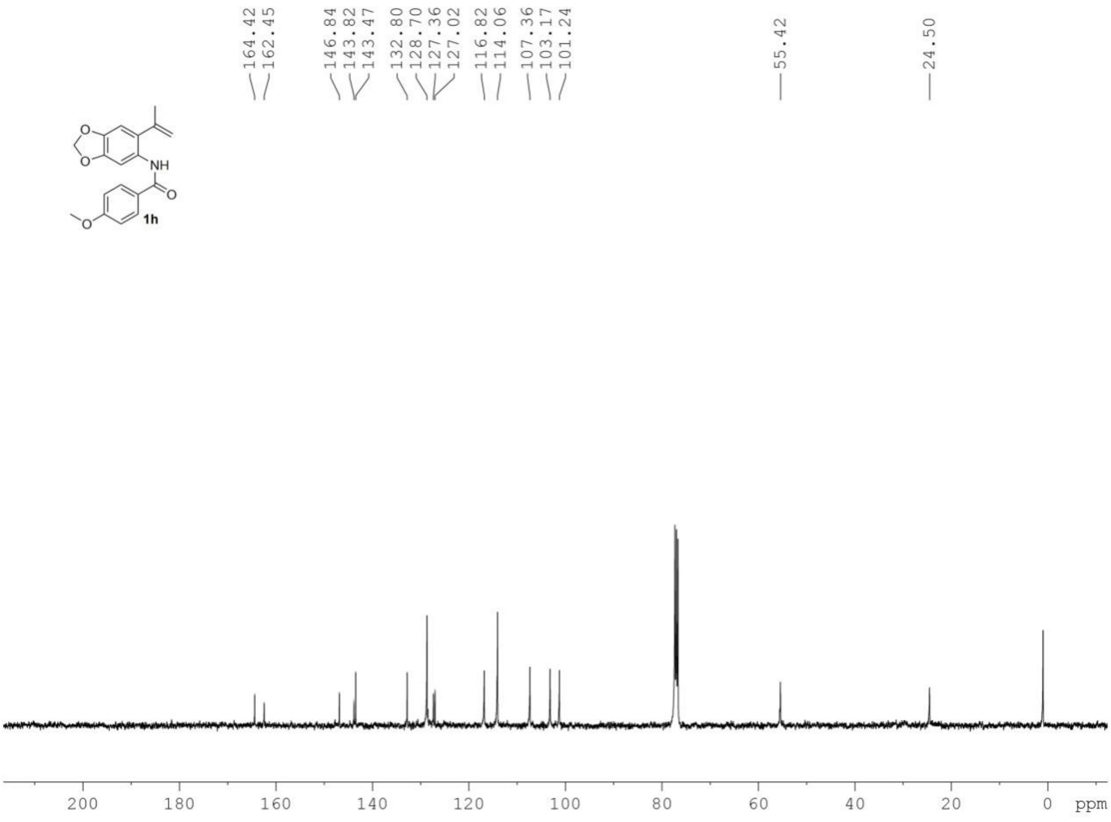

Figure S11. ^{13}C NMR spectrum of **1h**

5. ^{1}H and ^{13}C spectra of selenylated benzoxazines

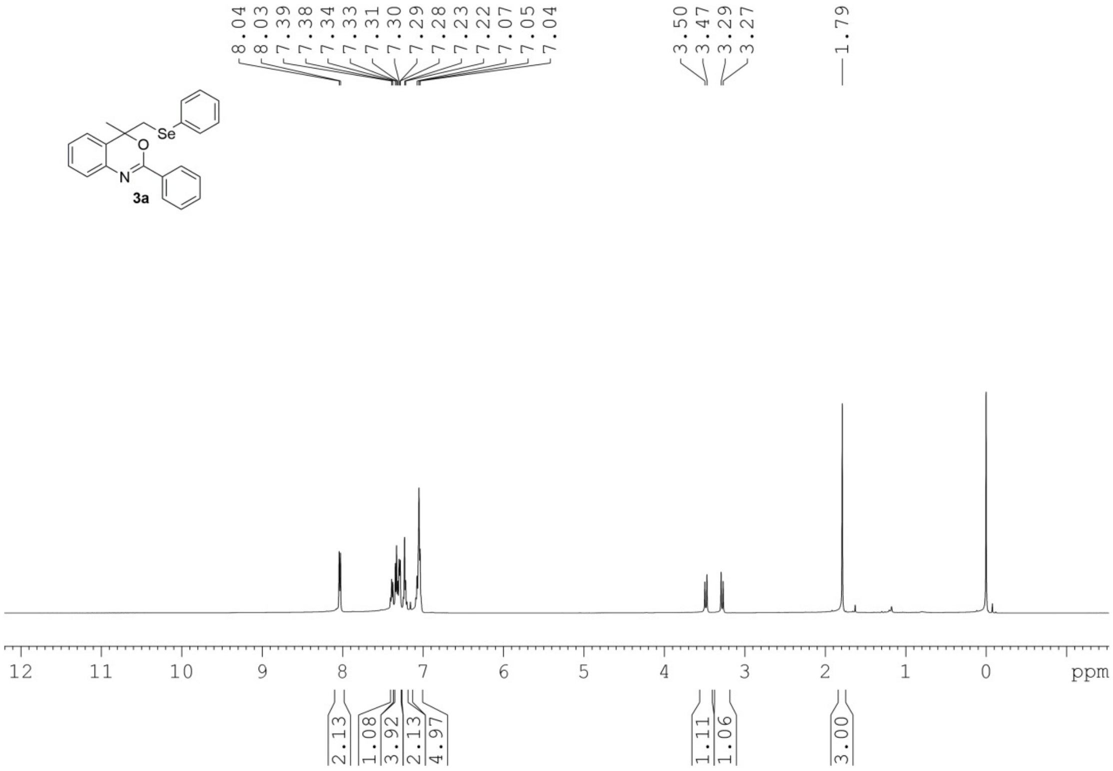

Figure S12. ^{1}H NMR spectrum of **3a**

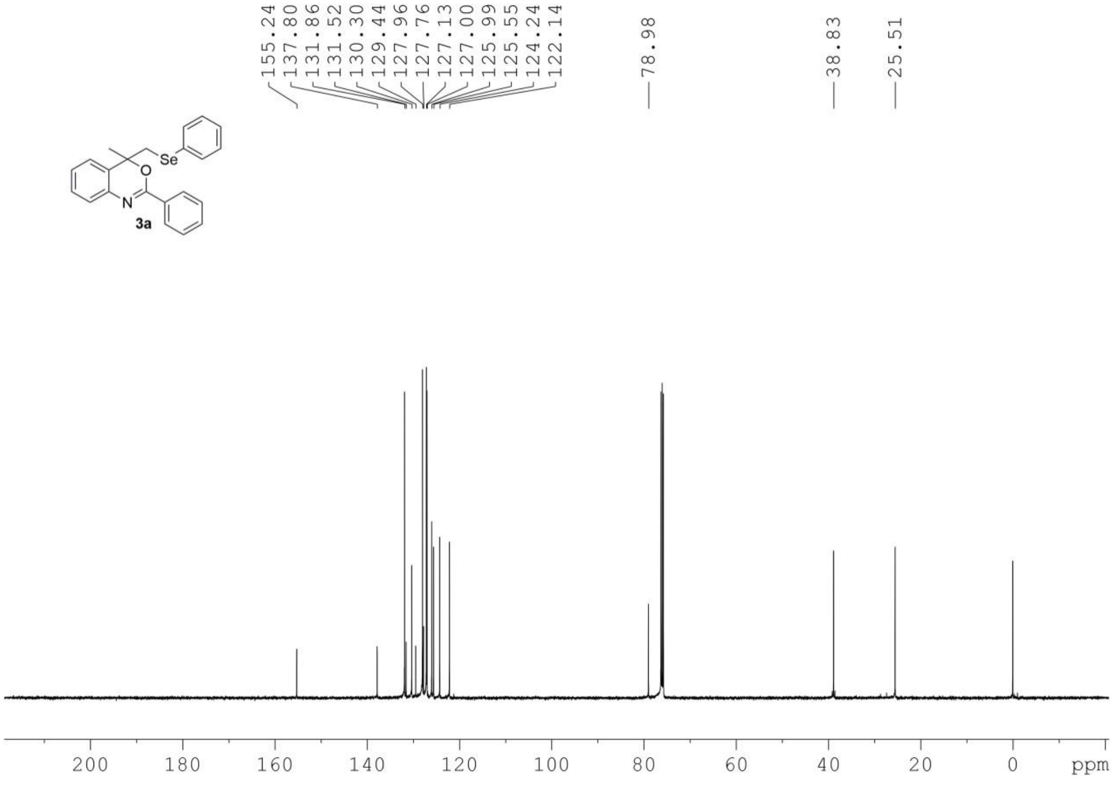

Figure S13. ^{13}C NMR spectrum of **3a**

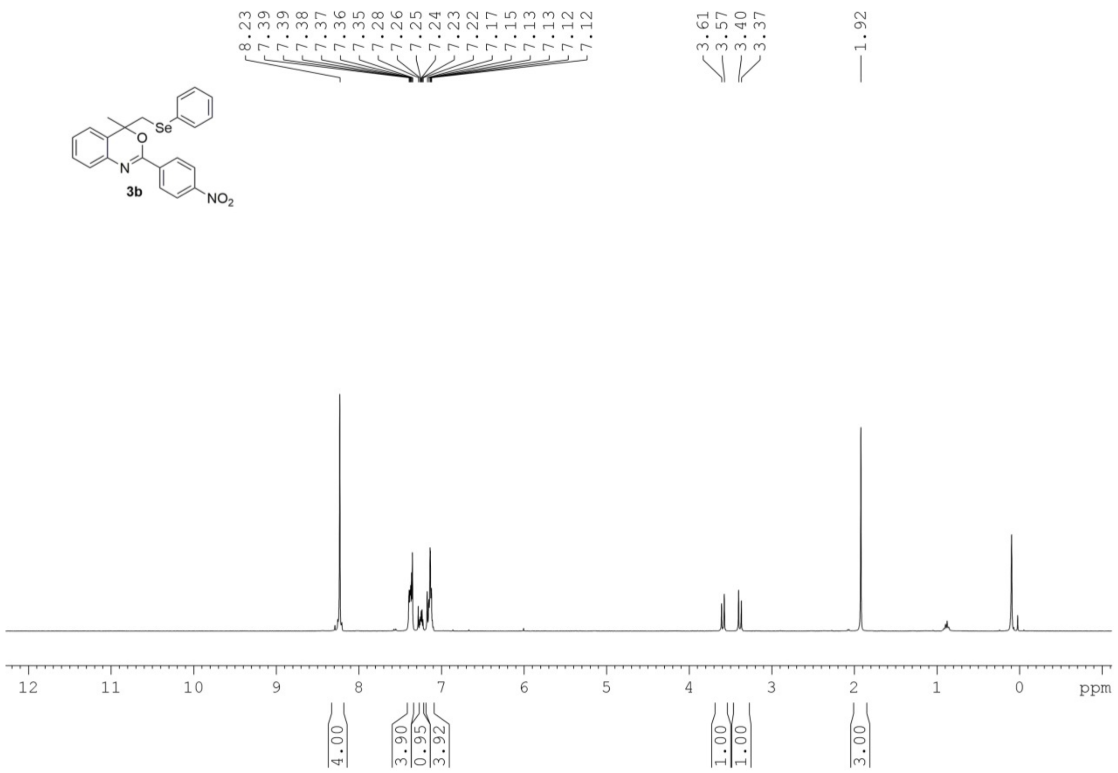

Figure S14. ^{1}H NMR spectrum of **3b**

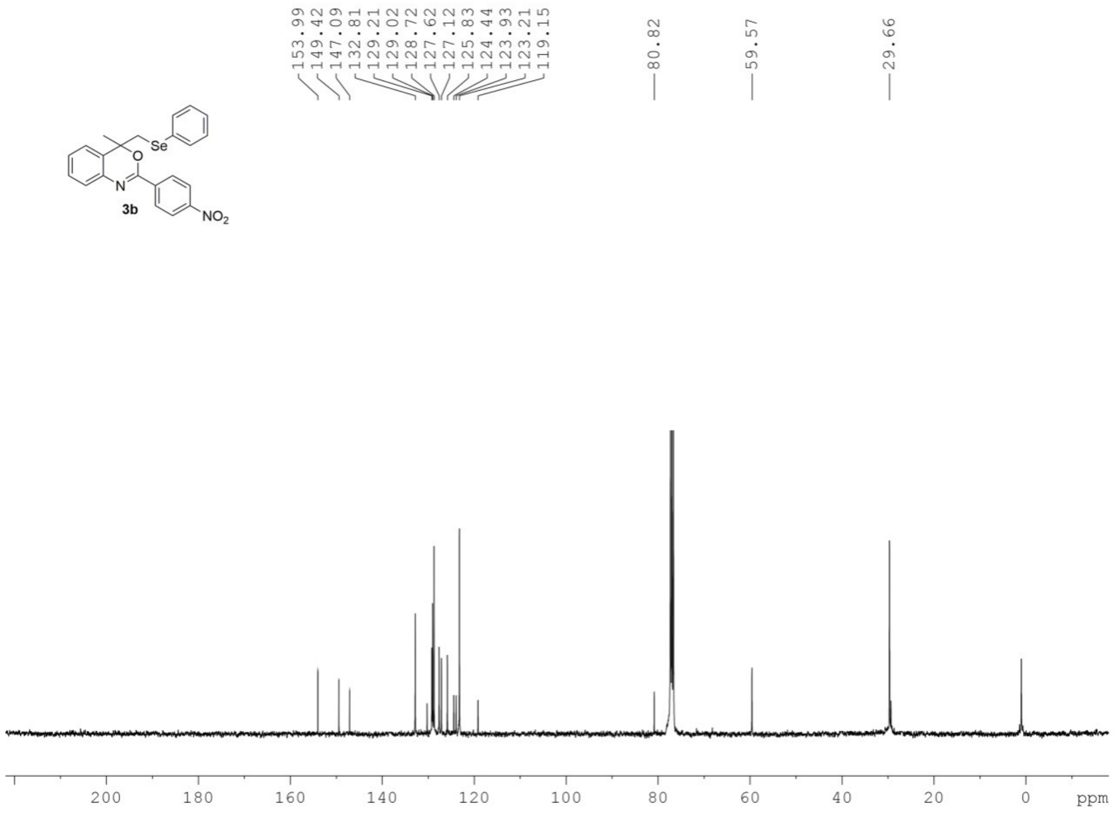

Figure S15. ^{13}C NMR spectrum of **3b**

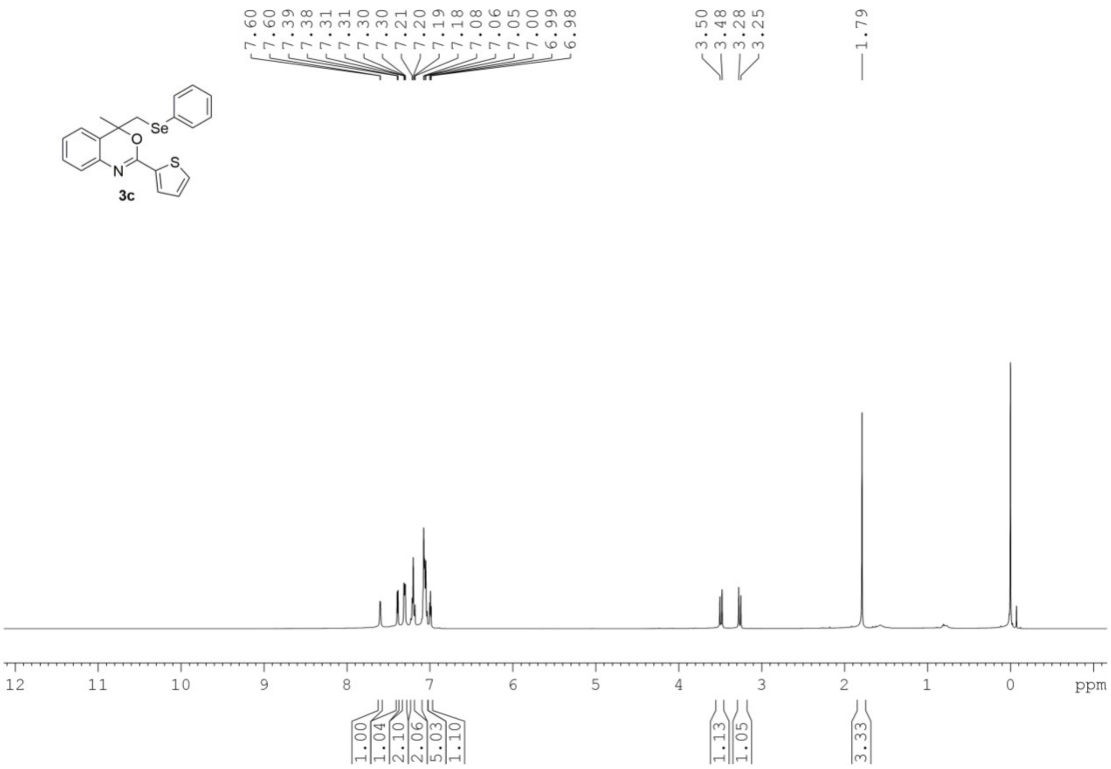

Figure 16. ^{1}H NMR spectrum of **3c**

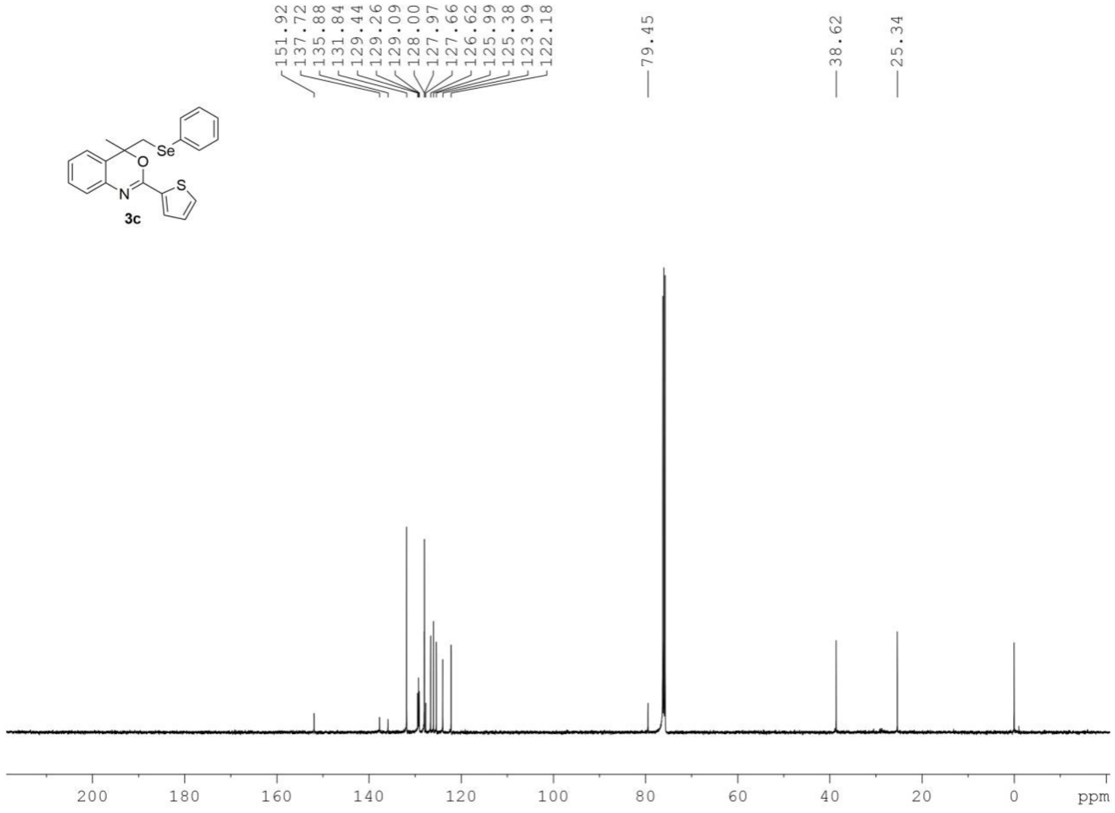

Figure S17. ^{13}C NMR spectrum of **3c**

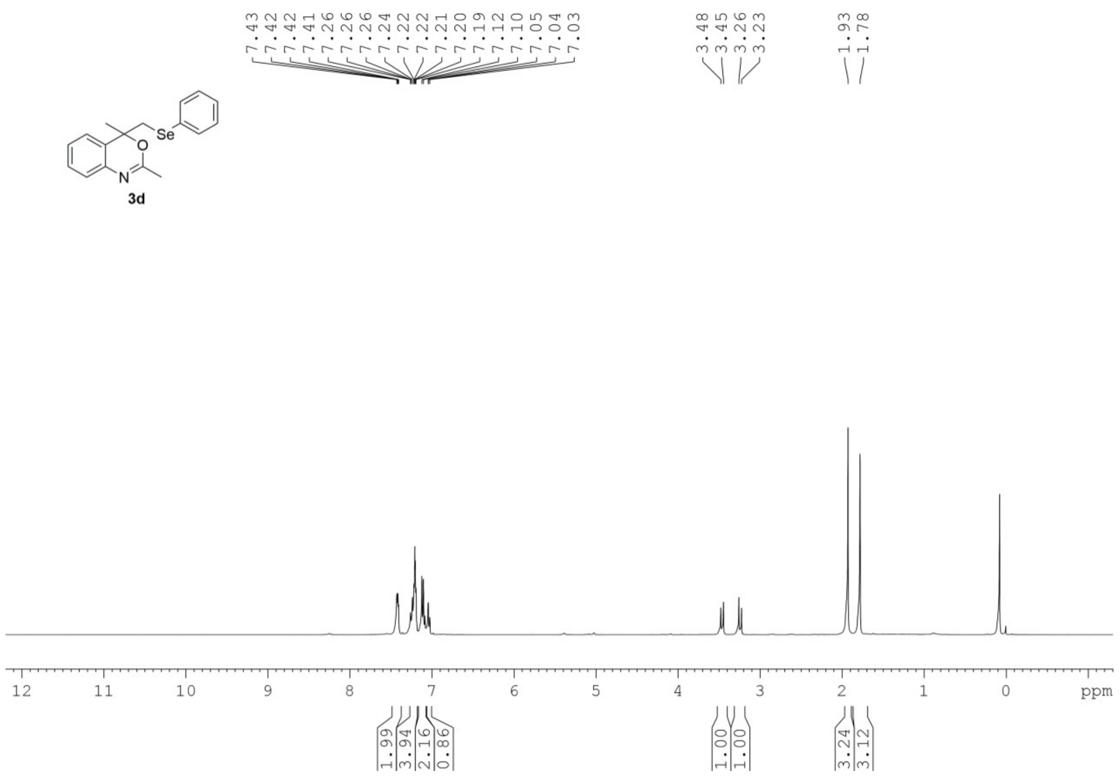

Figure 18. ^{1}H NMR spectrum of **3d**

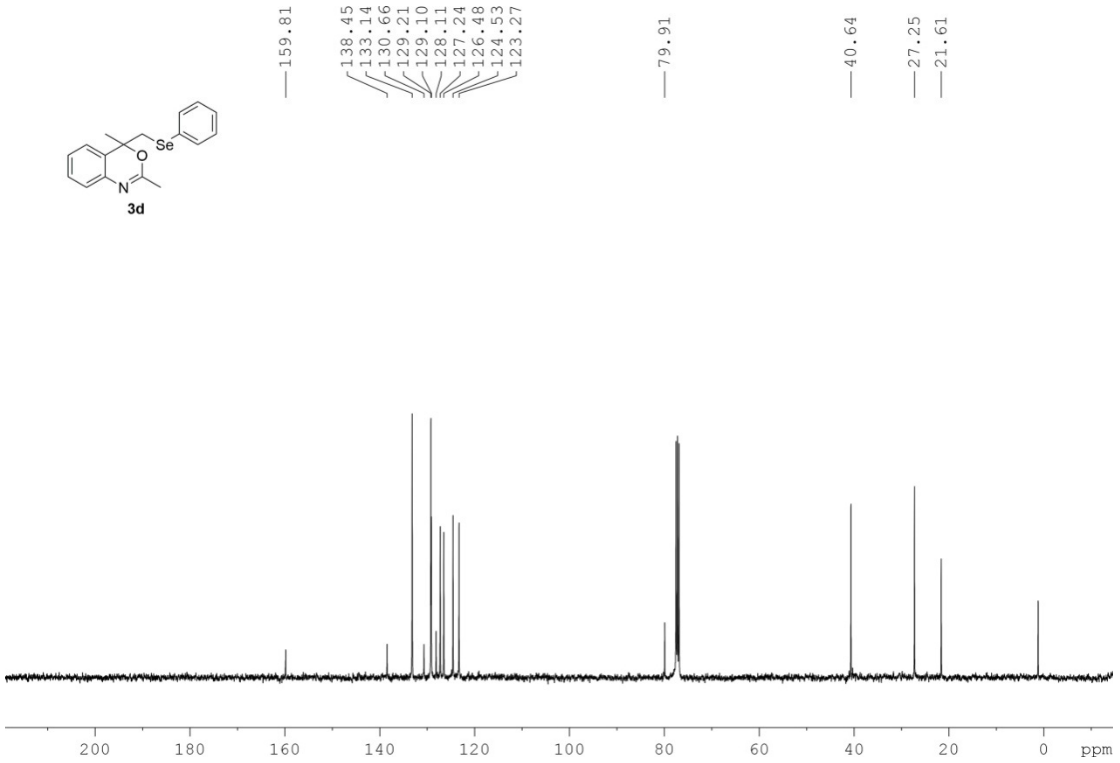

Figure S19. ^{13}C NMR spectrum of **3d**

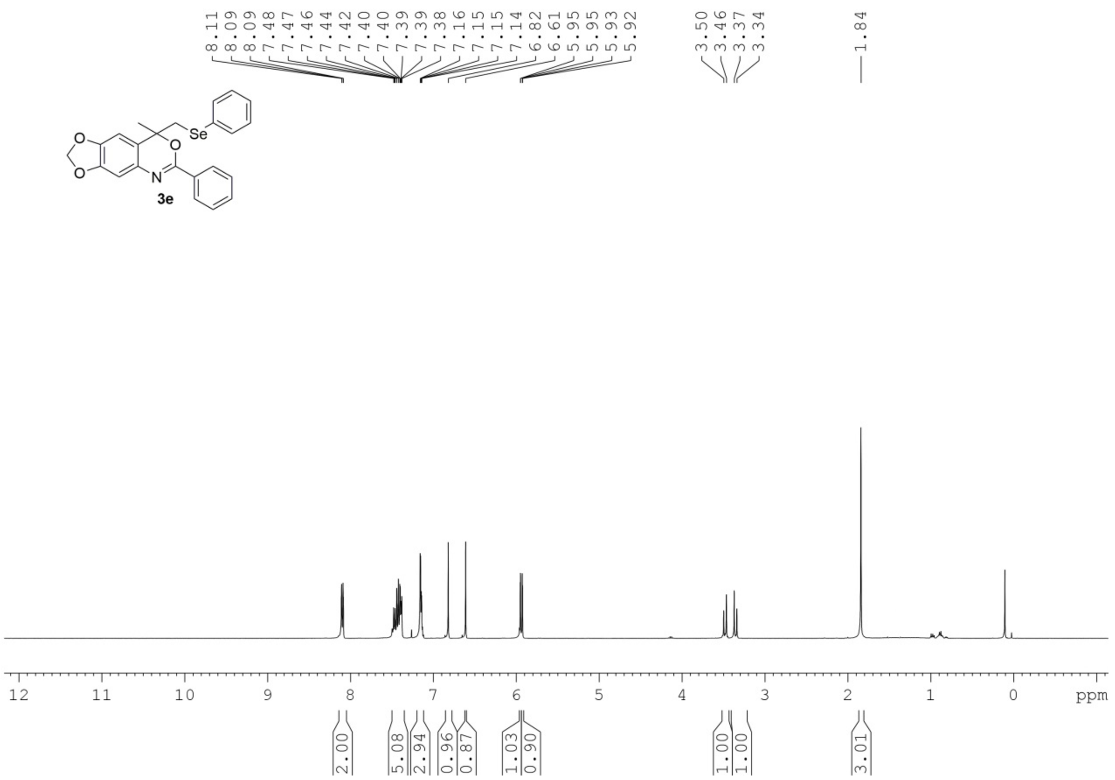

Figure S20. ^{1}H NMR spectrum of **3e**

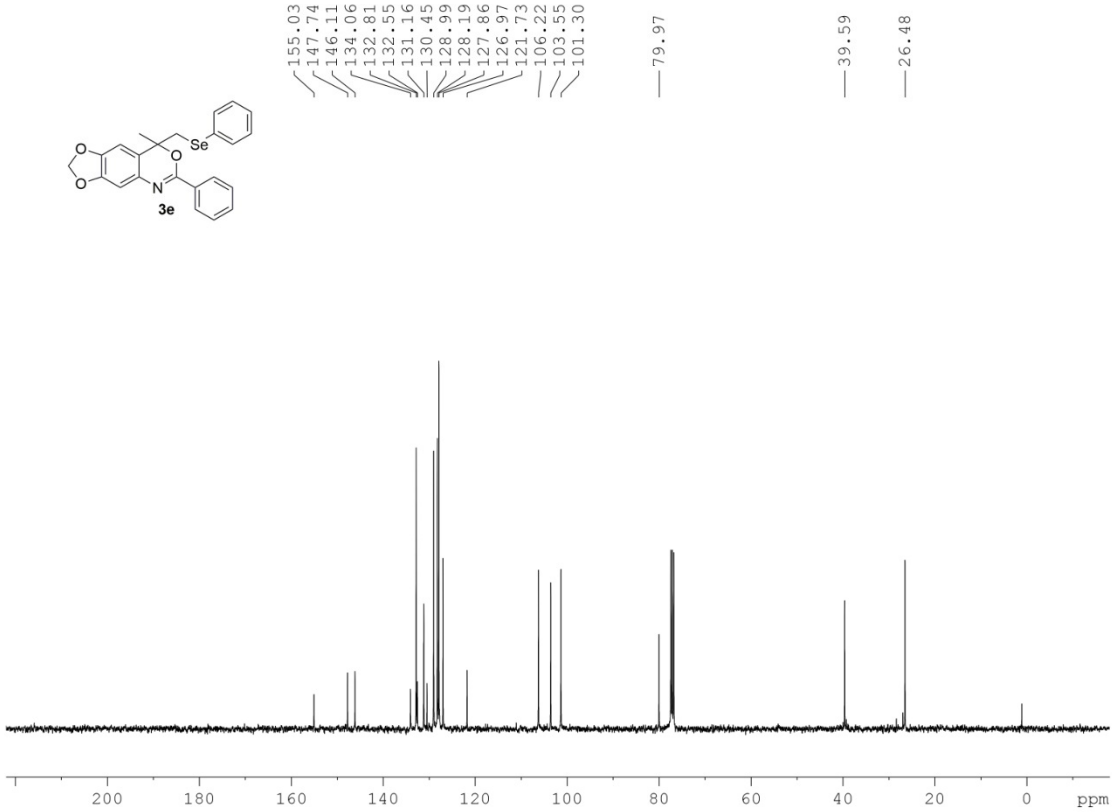

Figure S21. ^{13}C NMR spectrum of **3e**

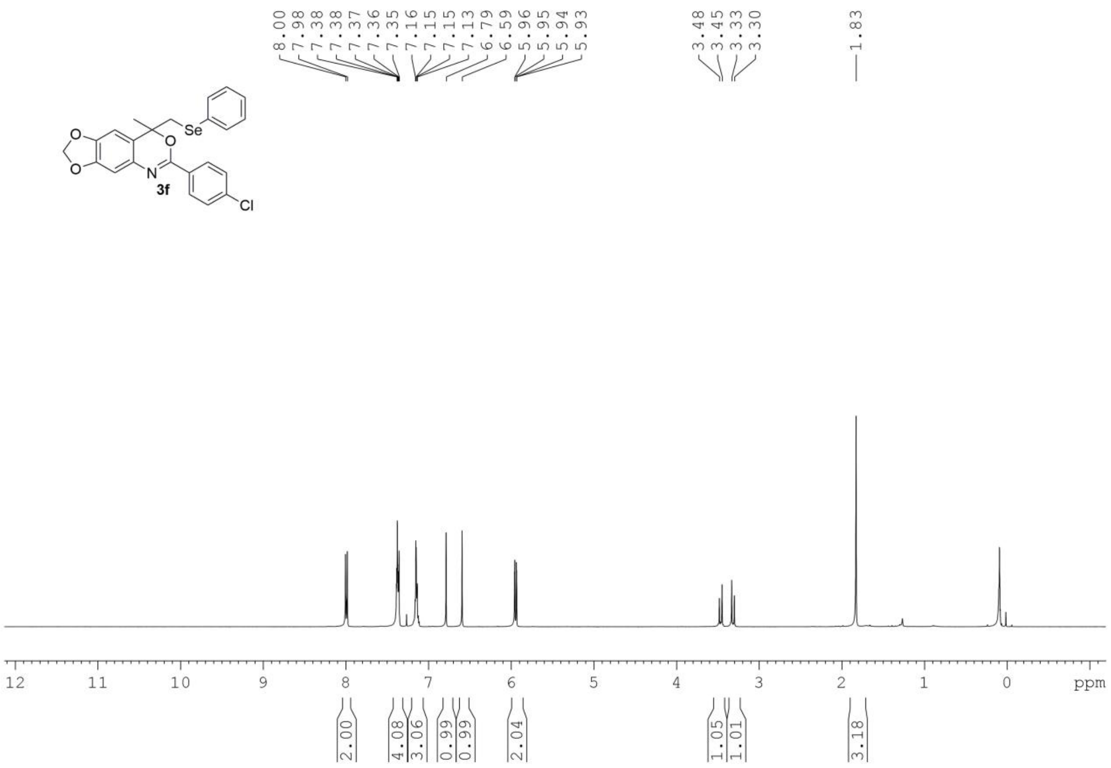

Figure S22. ^{1}H NMR spectrum of **3f**

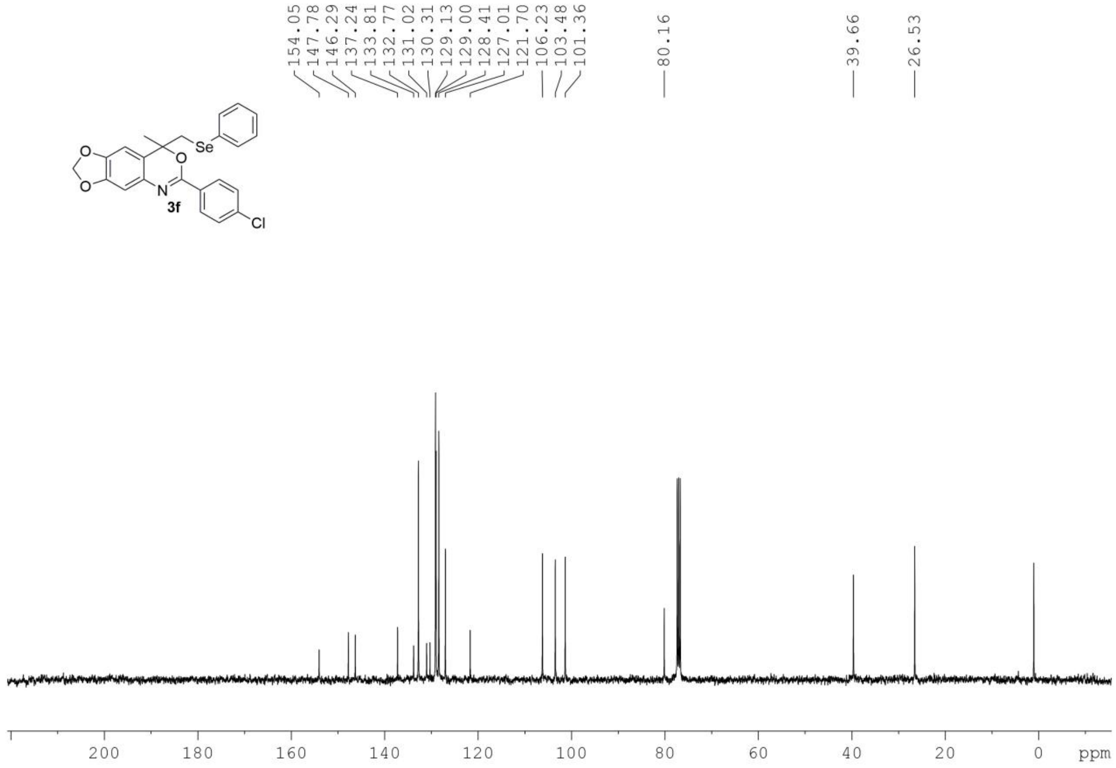

Figure S23. ^{13}C NMR spectrum of **3f**

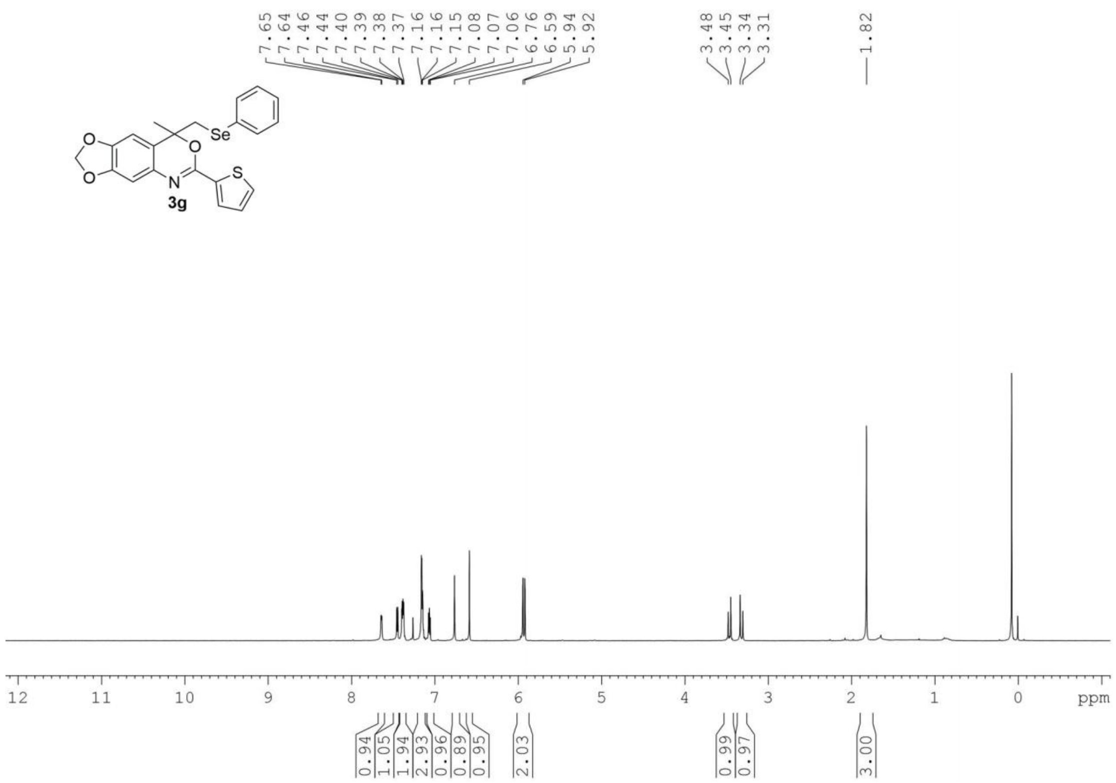

Figure S24. ^{1}H NMR spectrum of **3g**

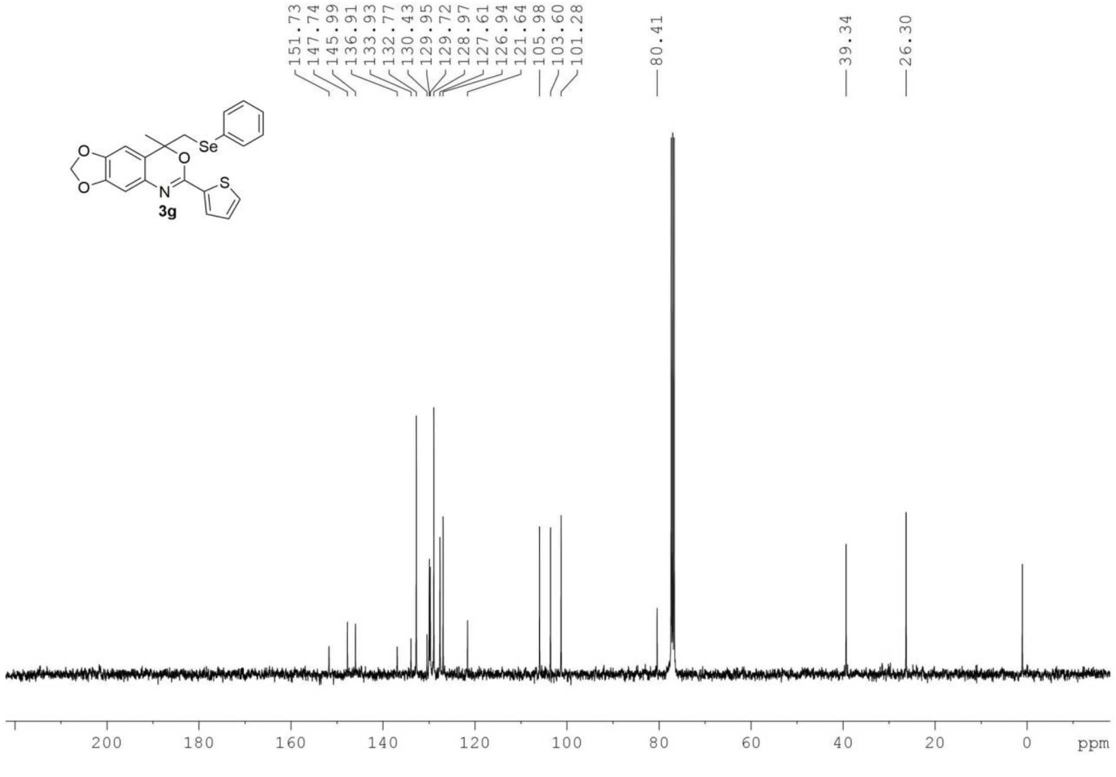

Figure S25. ^{13}C NMR spectrum of **3g**

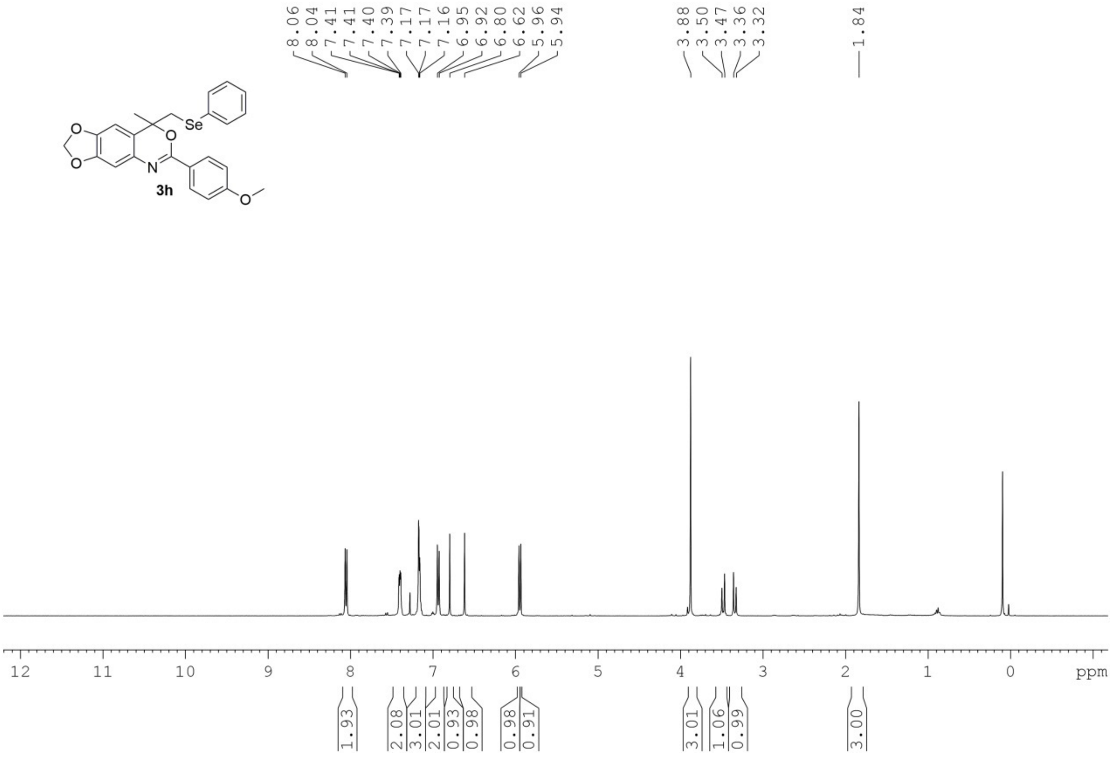

Figure S26. ^{1}H NMR spectrum of **3h**

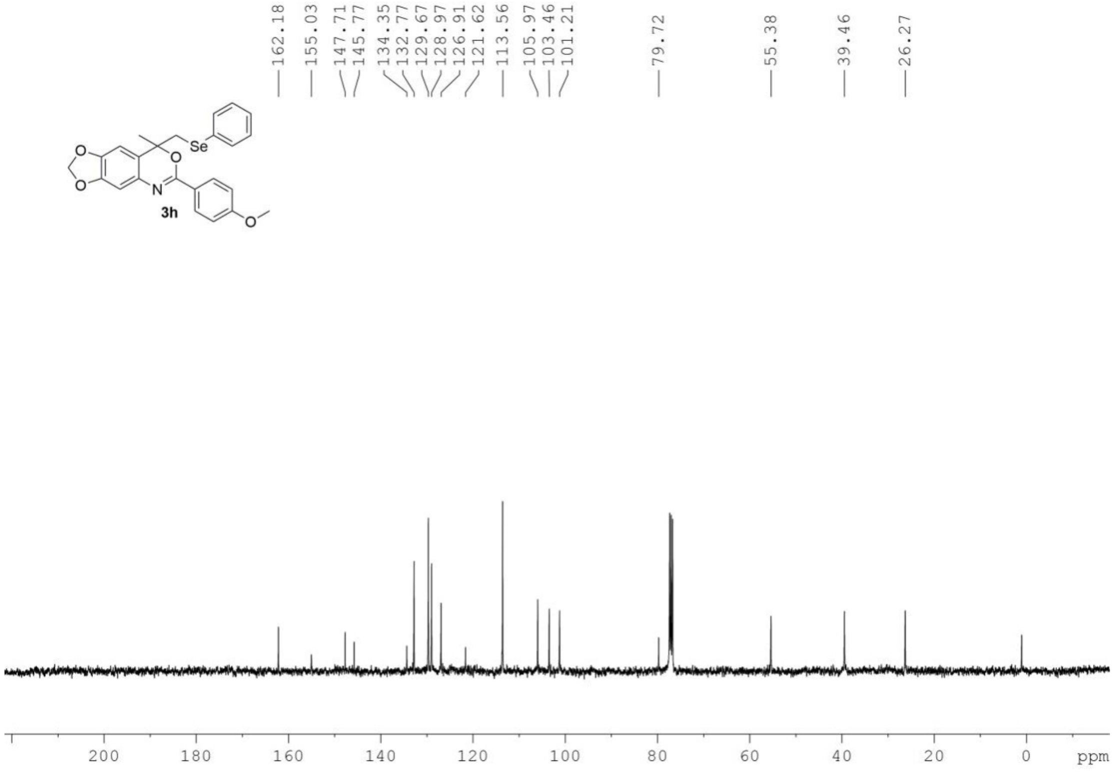

Figure S27. ^{13}C NMR spectrum of **3h**

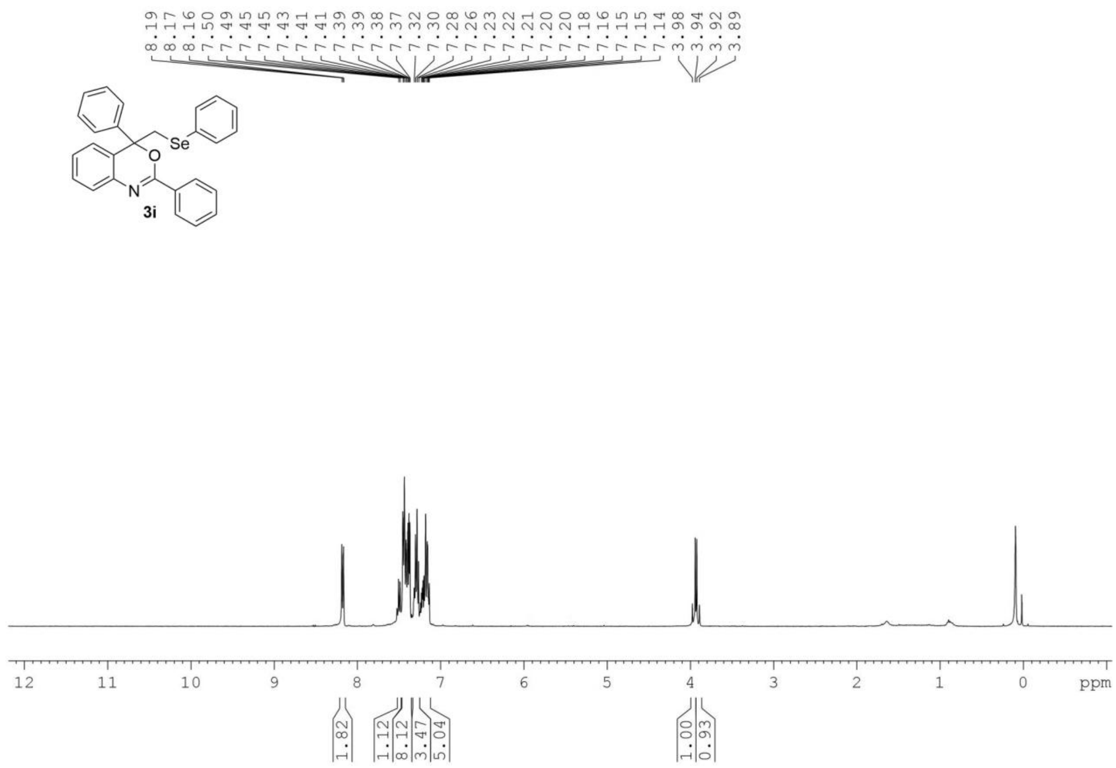

Figure S28. ^{1}H NMR spectrum of **3i**

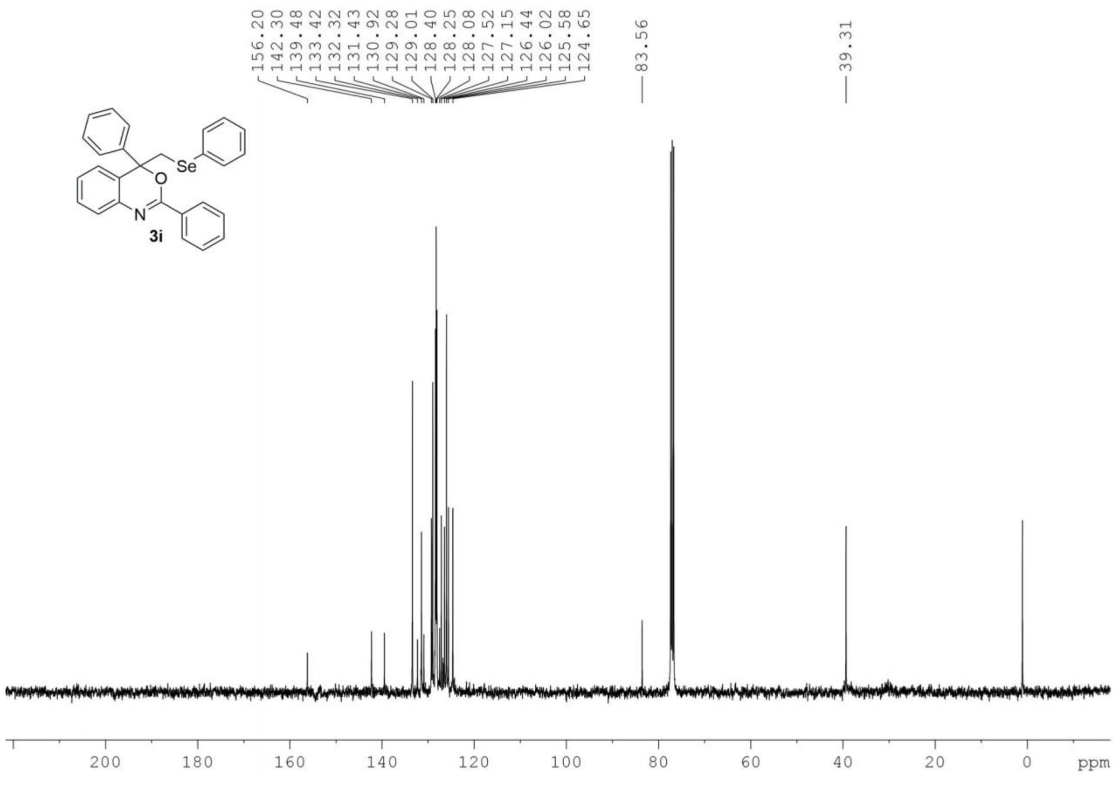

Figure S29. ^{13}C NMR spectrum of **3i**

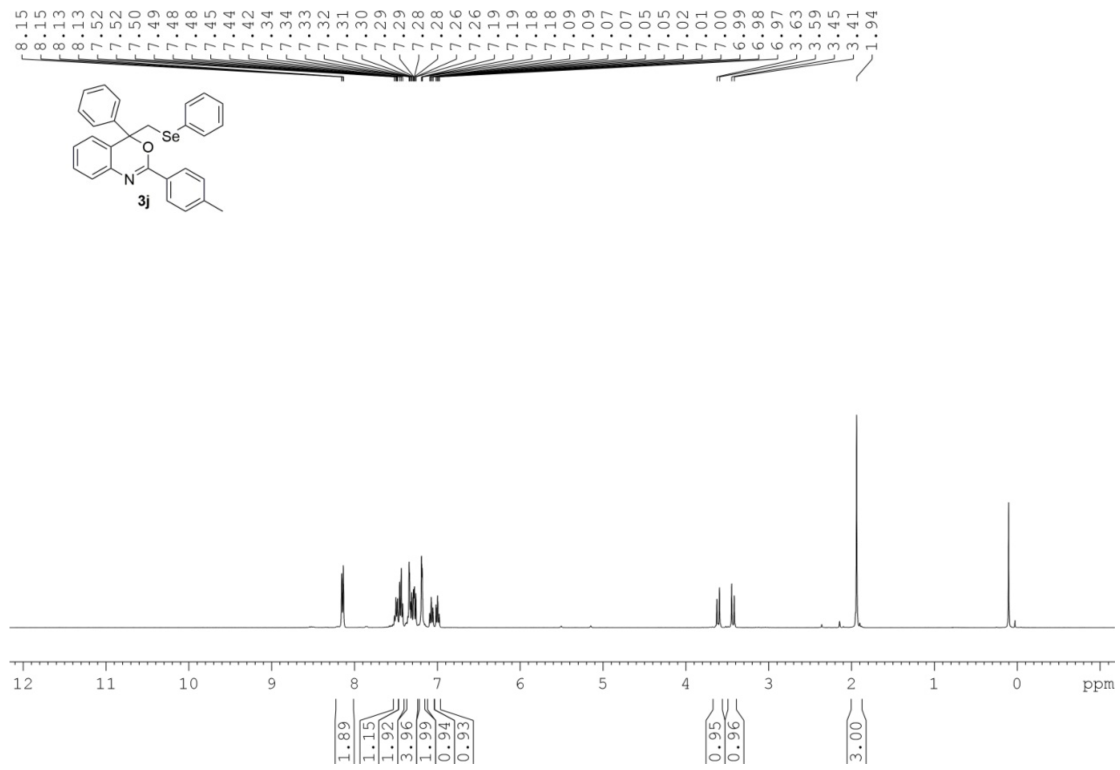

Figure S30. ^{1}H NMR spectrum of **3j**

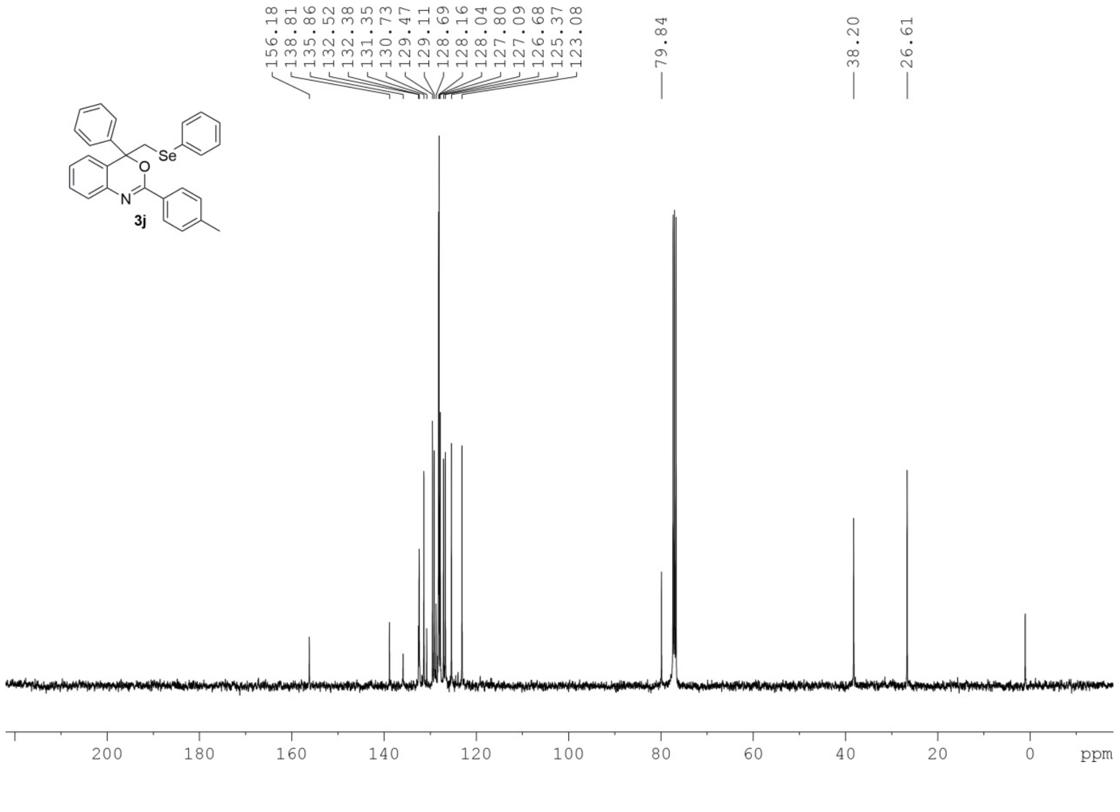

Figure S31. ^{13}C NMR spectrum of **3j**

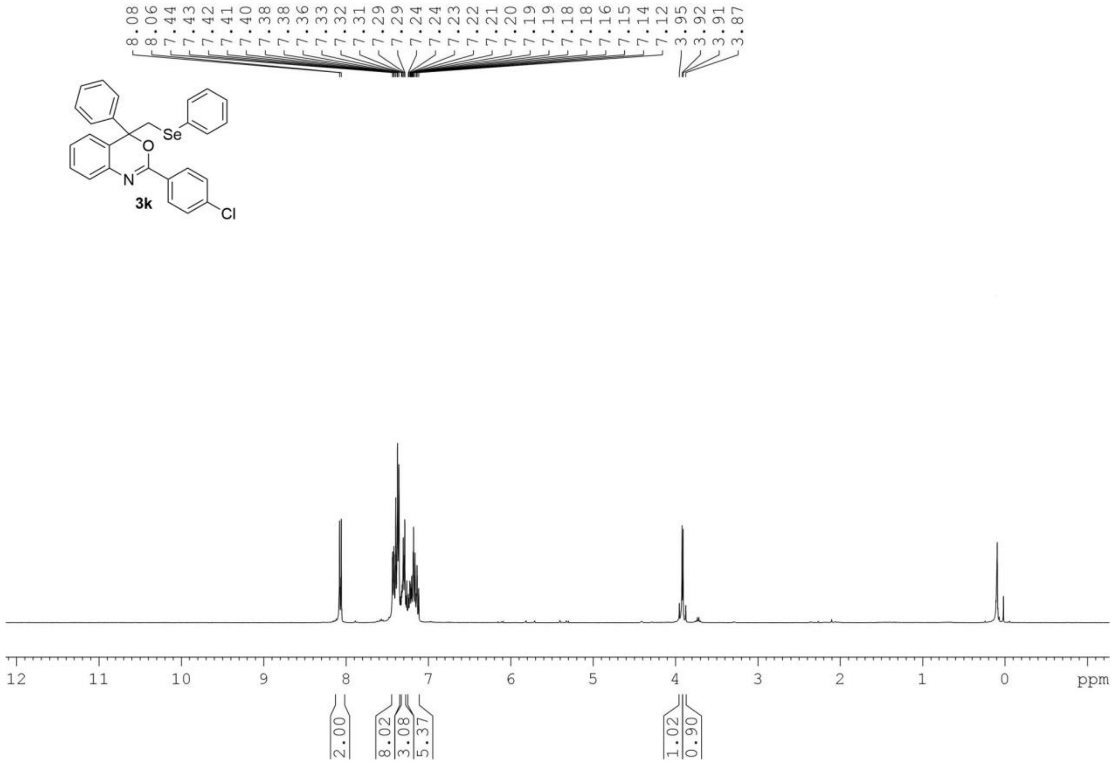

Figure S32. ^{1}H NMR spectrum of **3k**

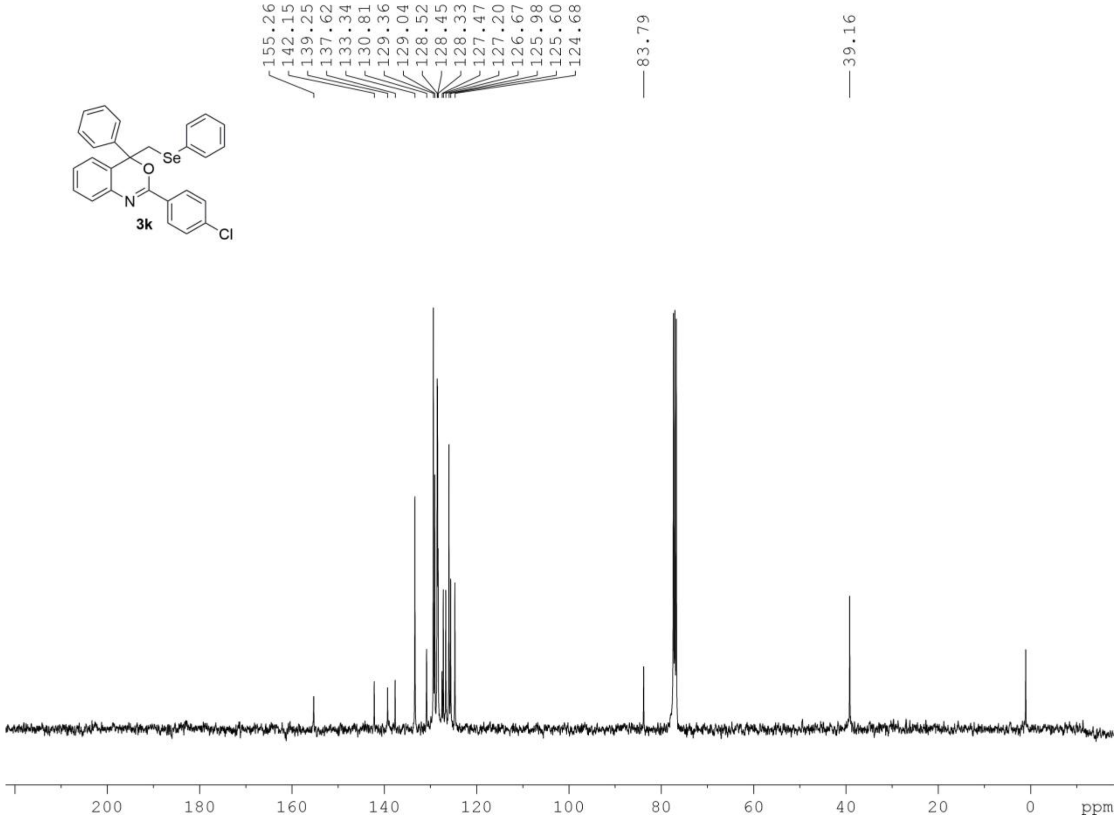

Figure S33. ^{13}C NMR spectrum of **3k**

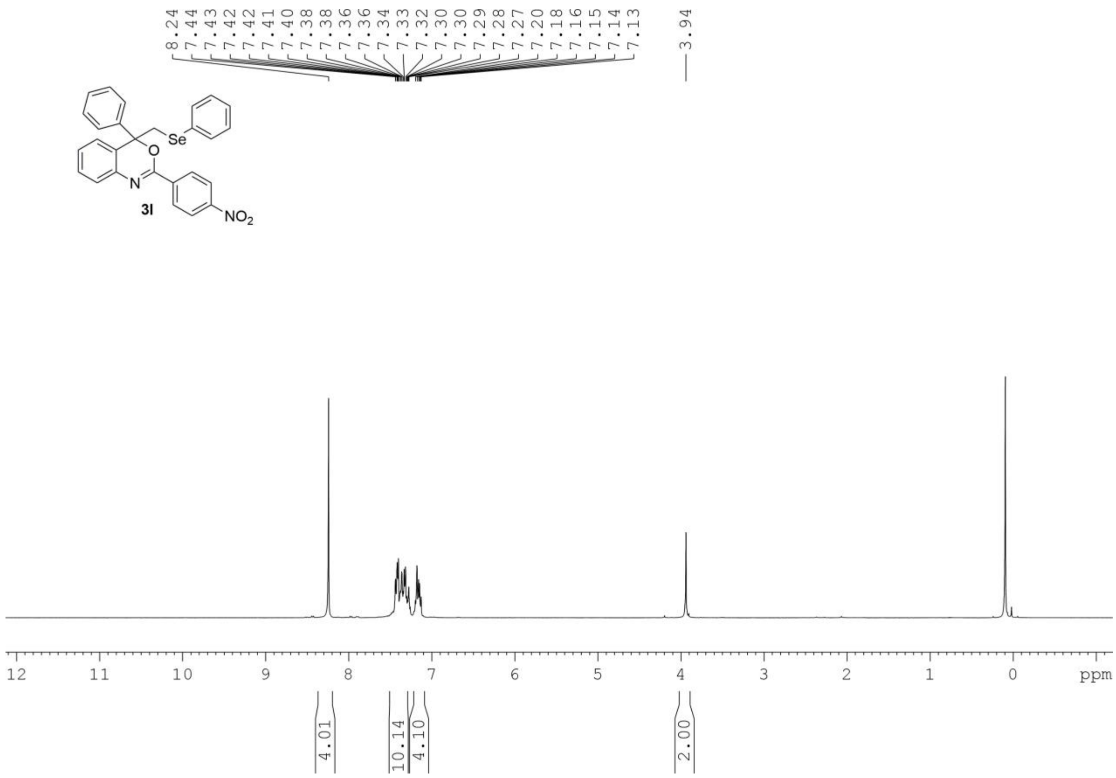

Figure S34. ^{1}H NMR spectrum of **3l**

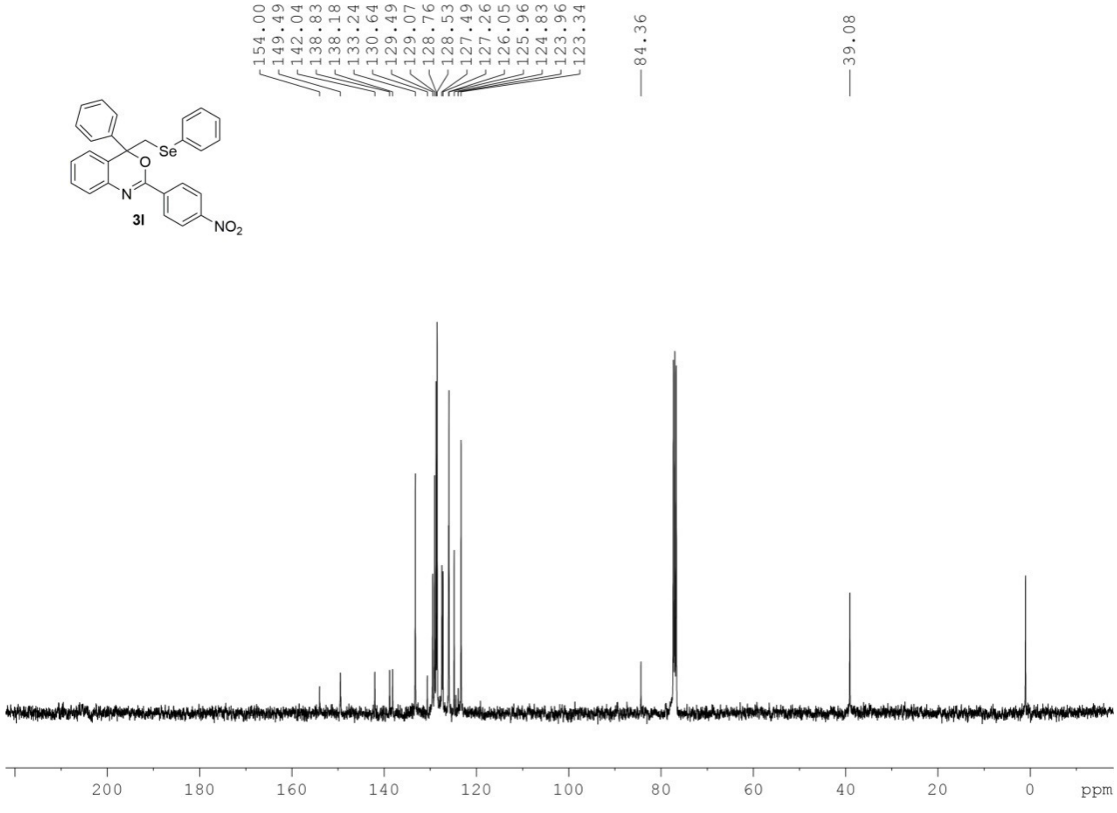

Figure S35. ^{13}C NMR spectrum of **3l**

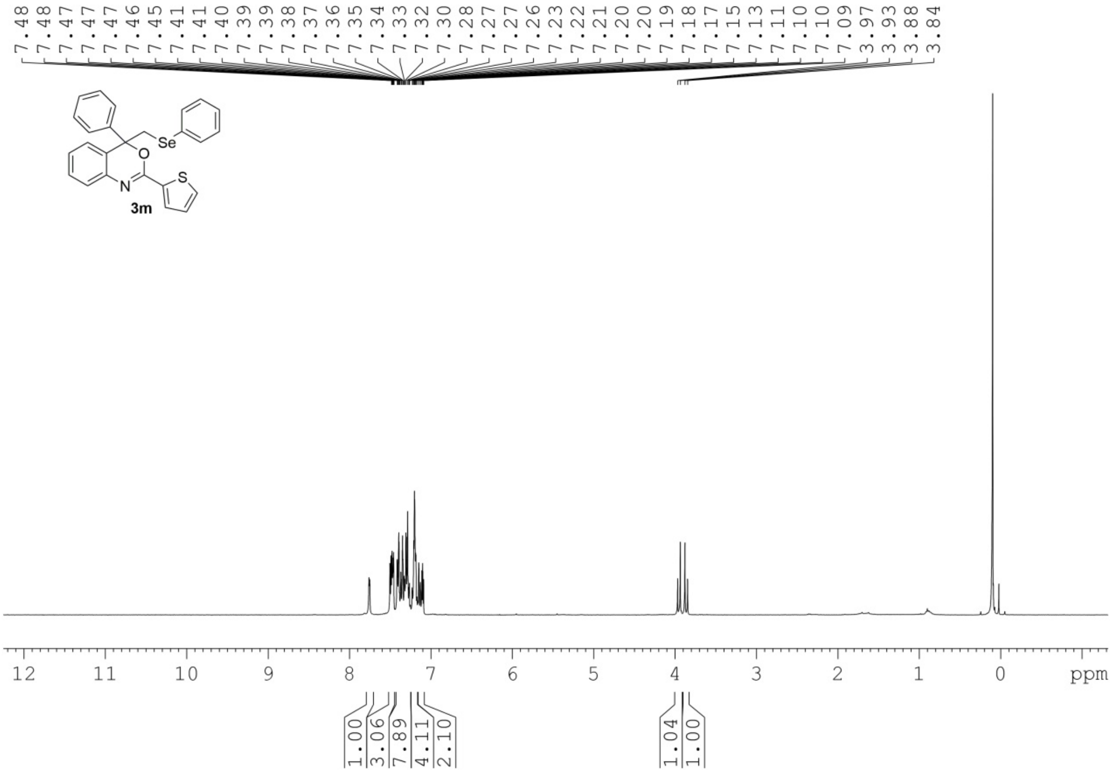

Figure S36. ^{1}H NMR spectrum of **3m**

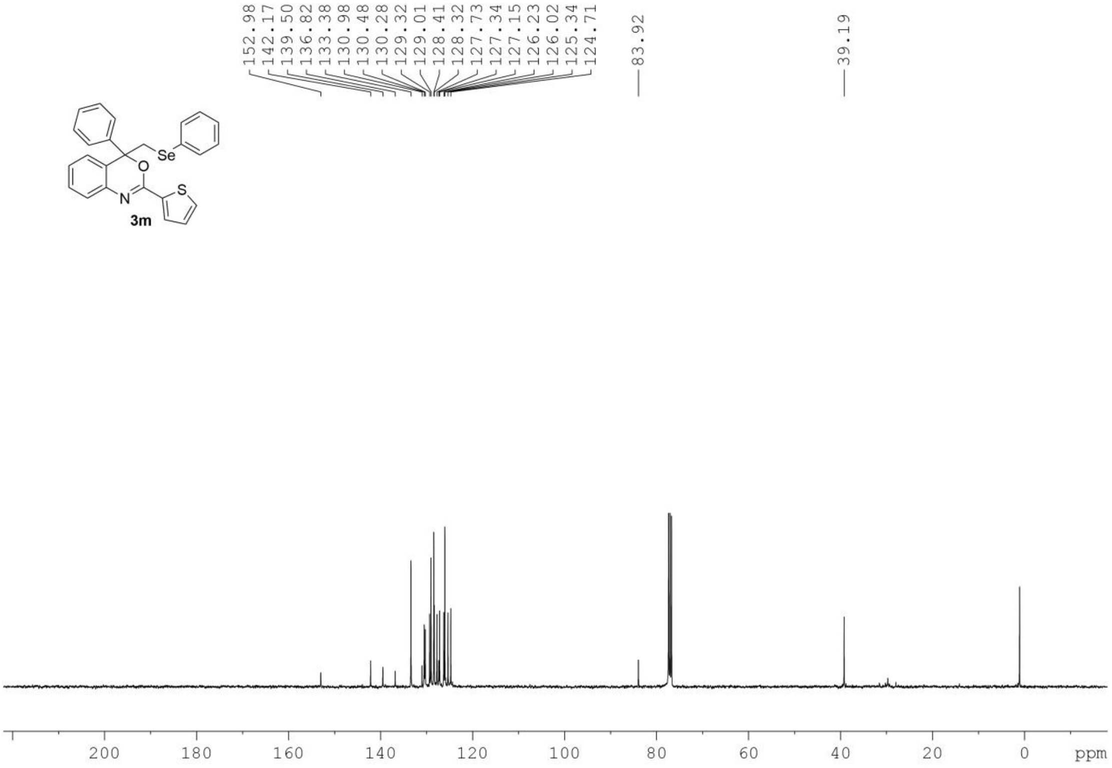

Figure S37. ^{13}C NMR spectrum of **3m**

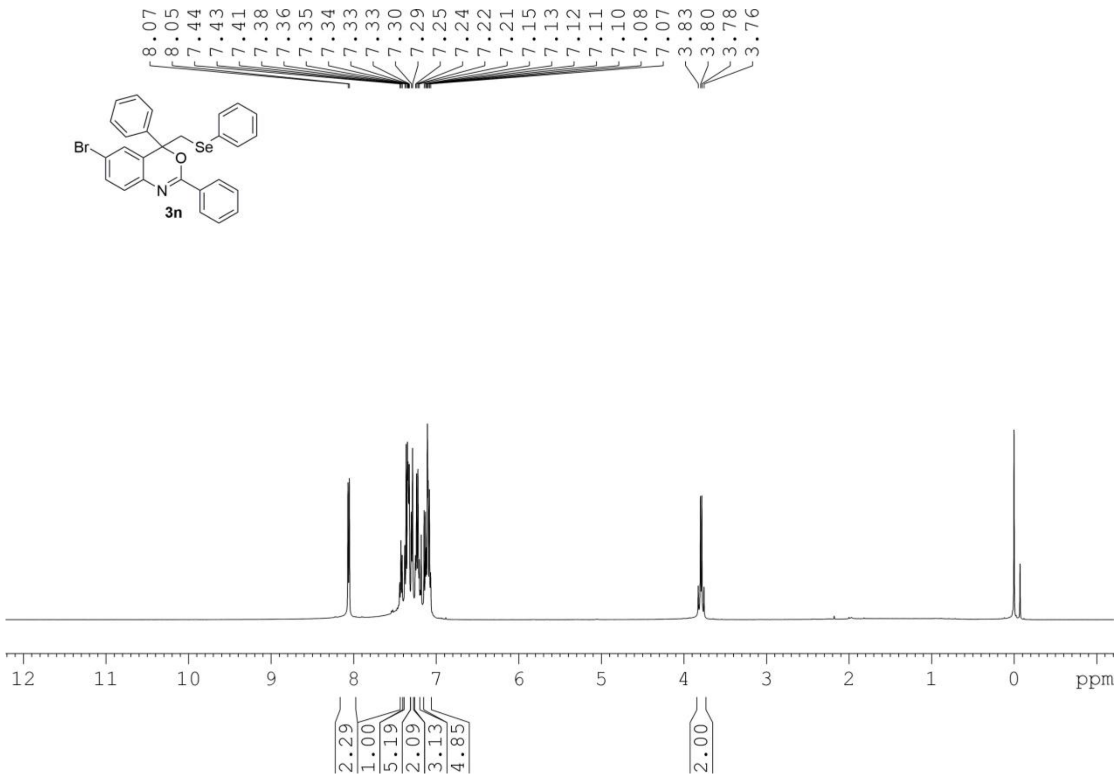

Figure S38. ^{1}H NMR spectrum of **3n**

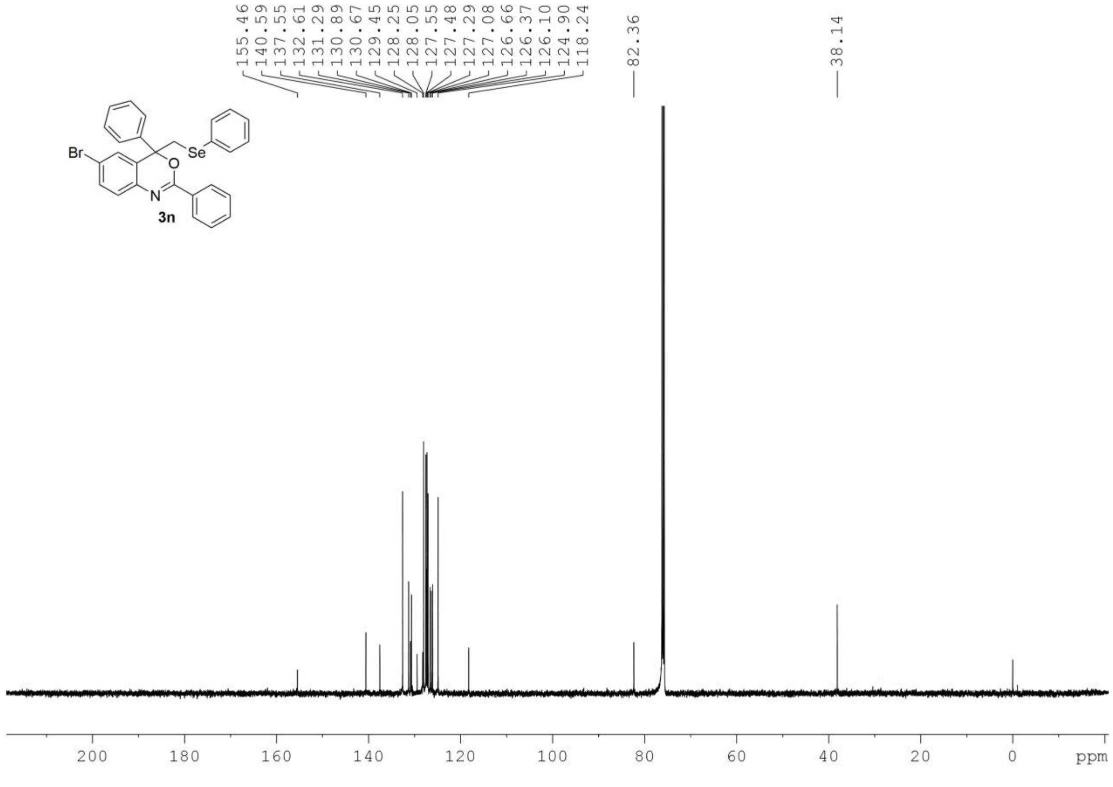

Figure S39. ^{13}C NMR spectrum of **3n**

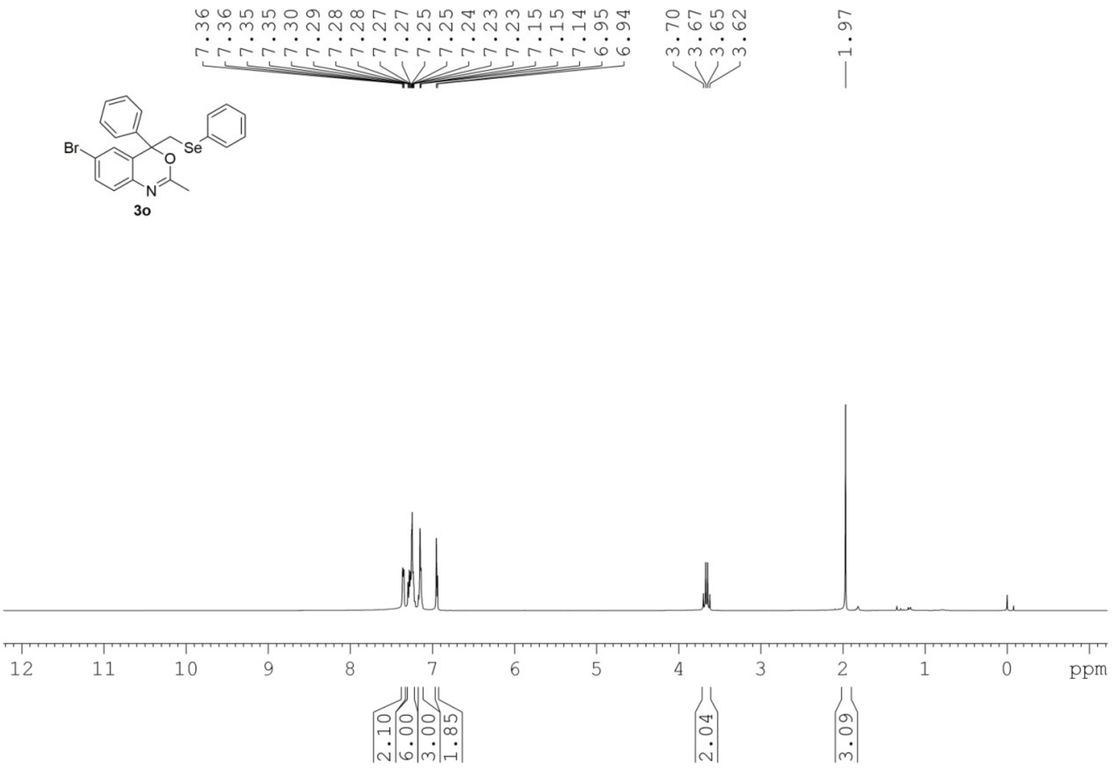

Figure S40. ^{1}H NMR spectrum of **3o**

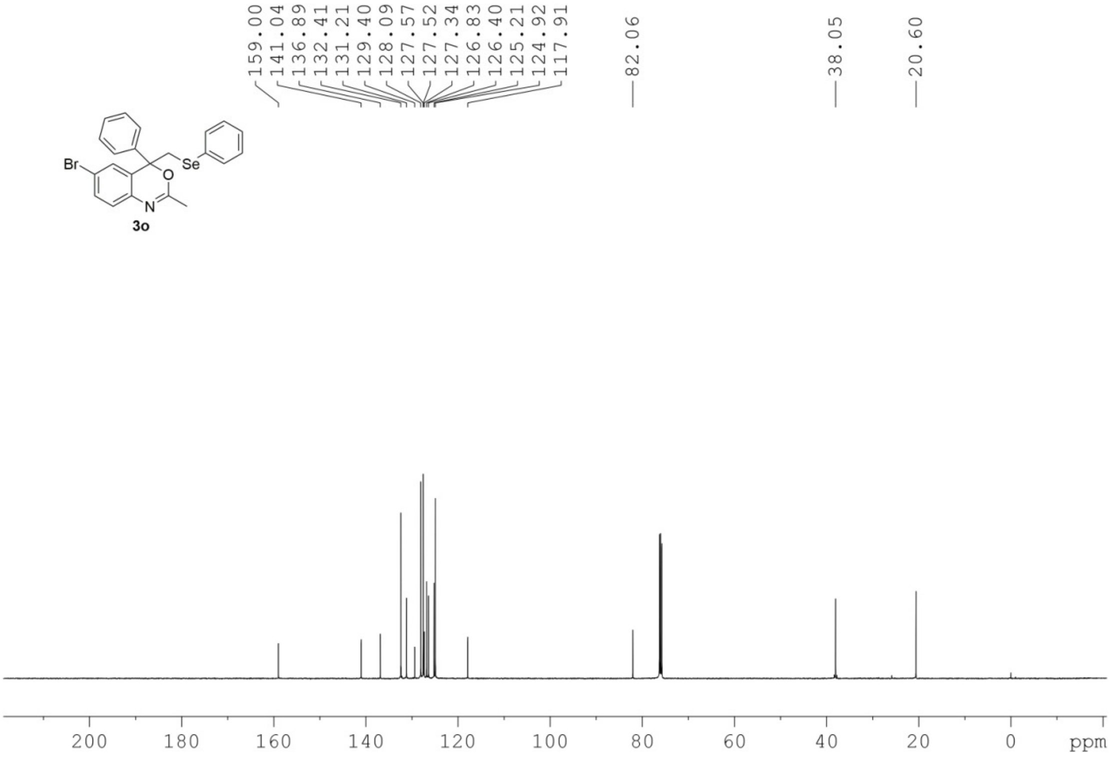

Figure S41. ^{13}C NMR spectrum of **3o**

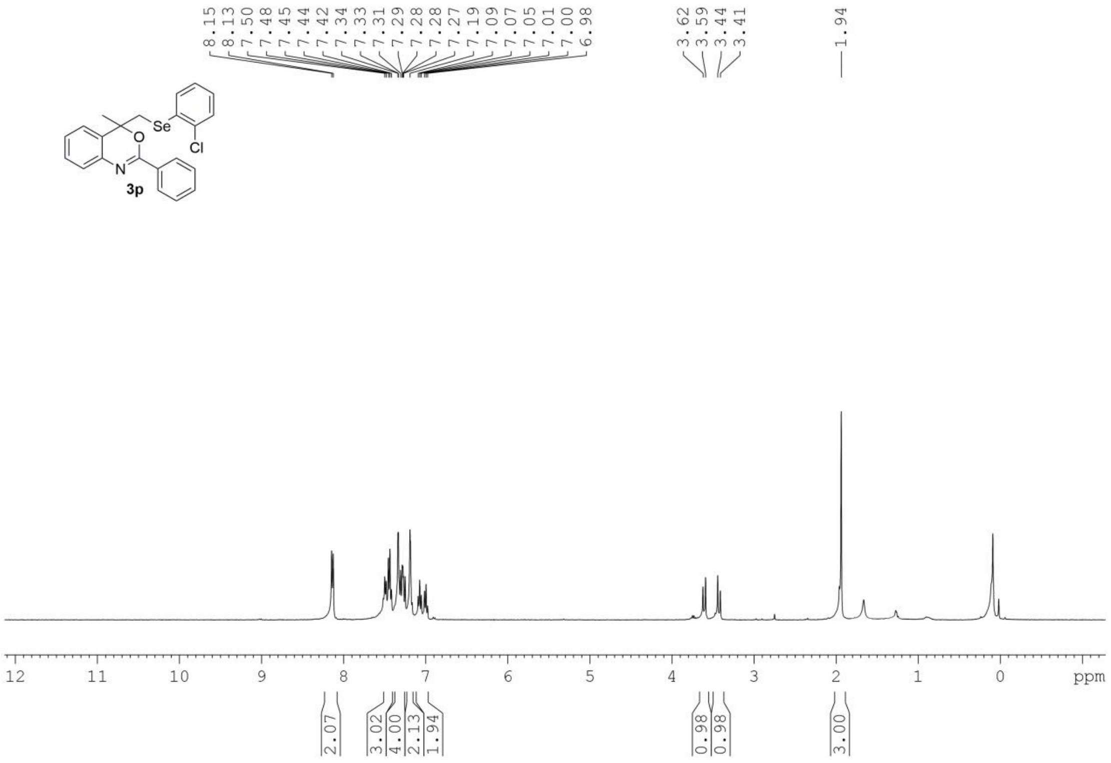

Figure S42. ^{1}H NMR spectrum of **3p**

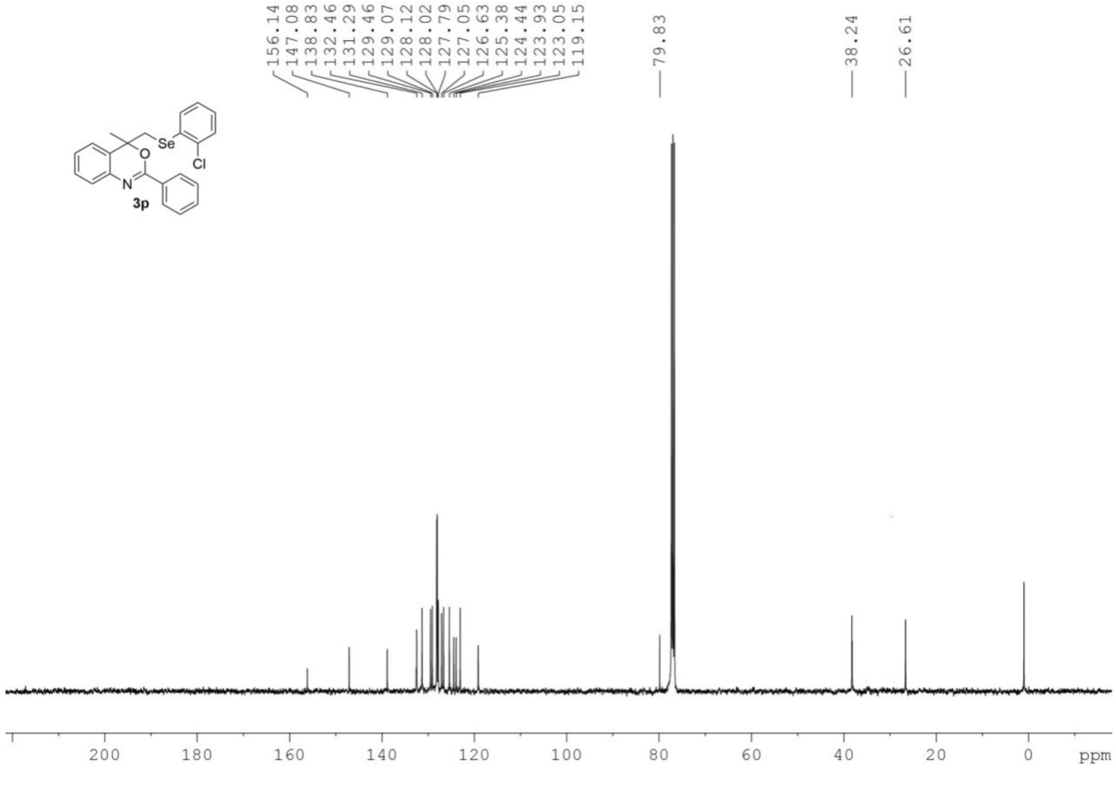

Figure S43. ^{13}C NMR spectrum of **3p**

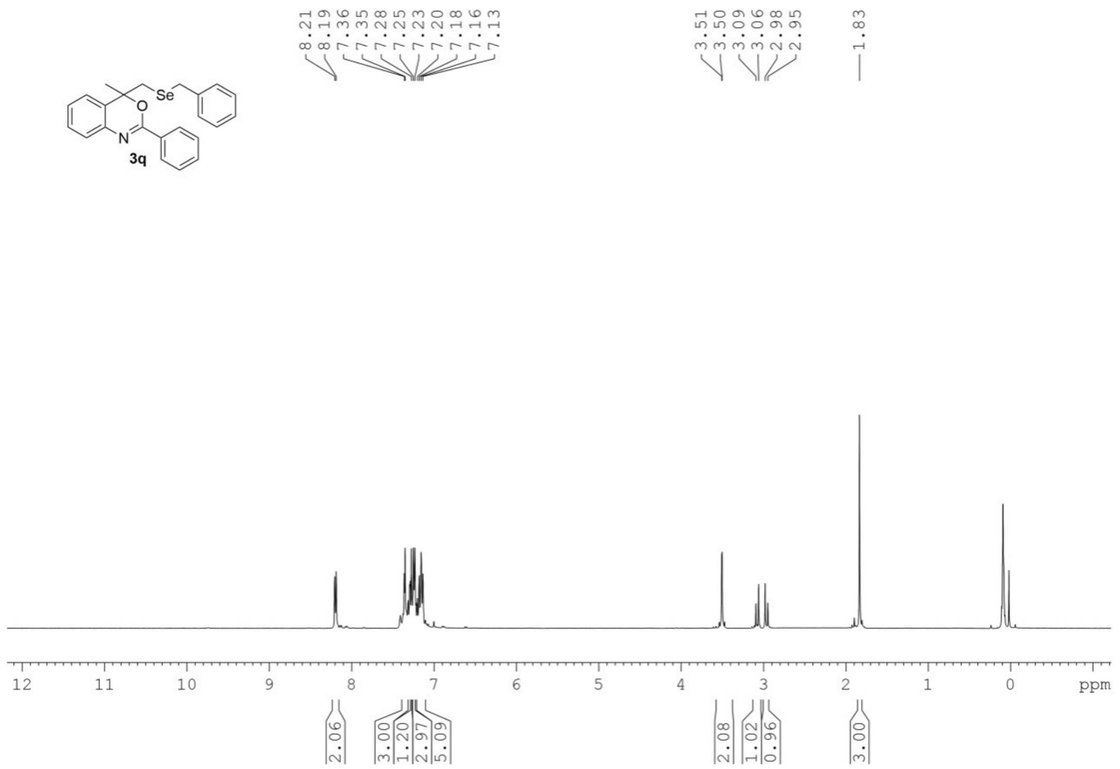

Figure S44. ^{1}H NMR spectrum of **3q**

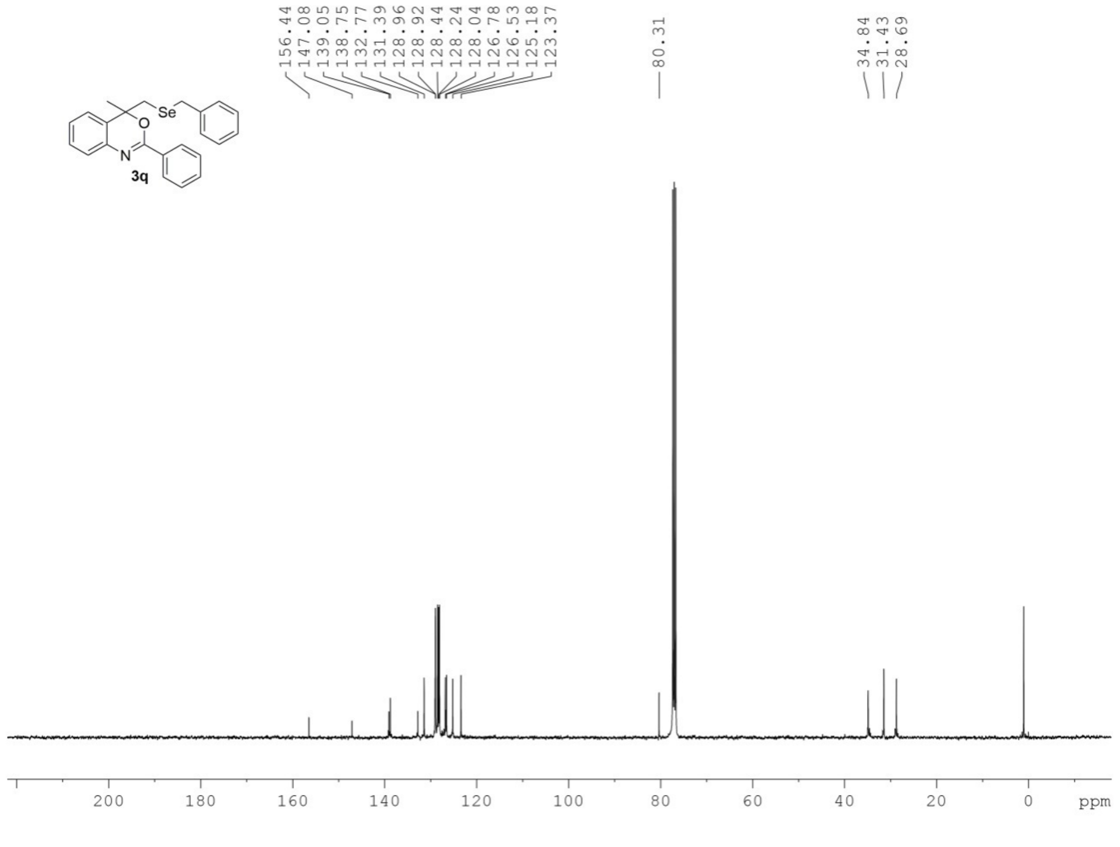

Figure S45. ^{13}C NMR spectrum of **3q**

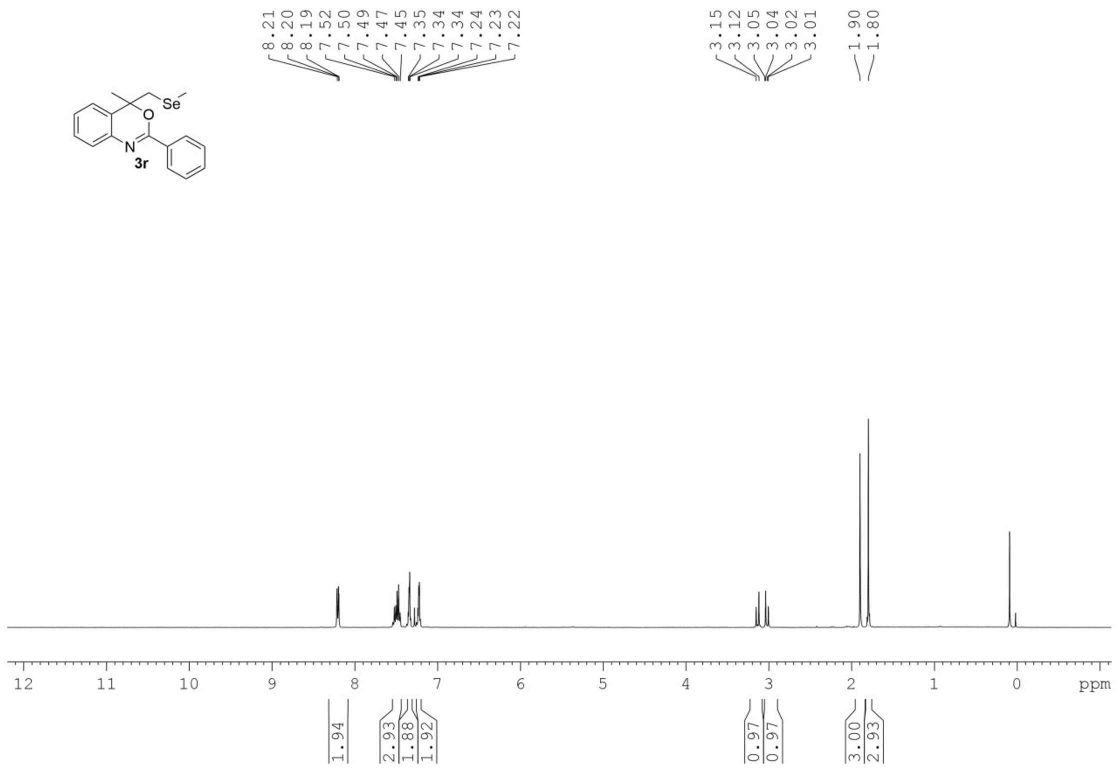

Figure S46. ^{1}H NMR spectrum of **3r**

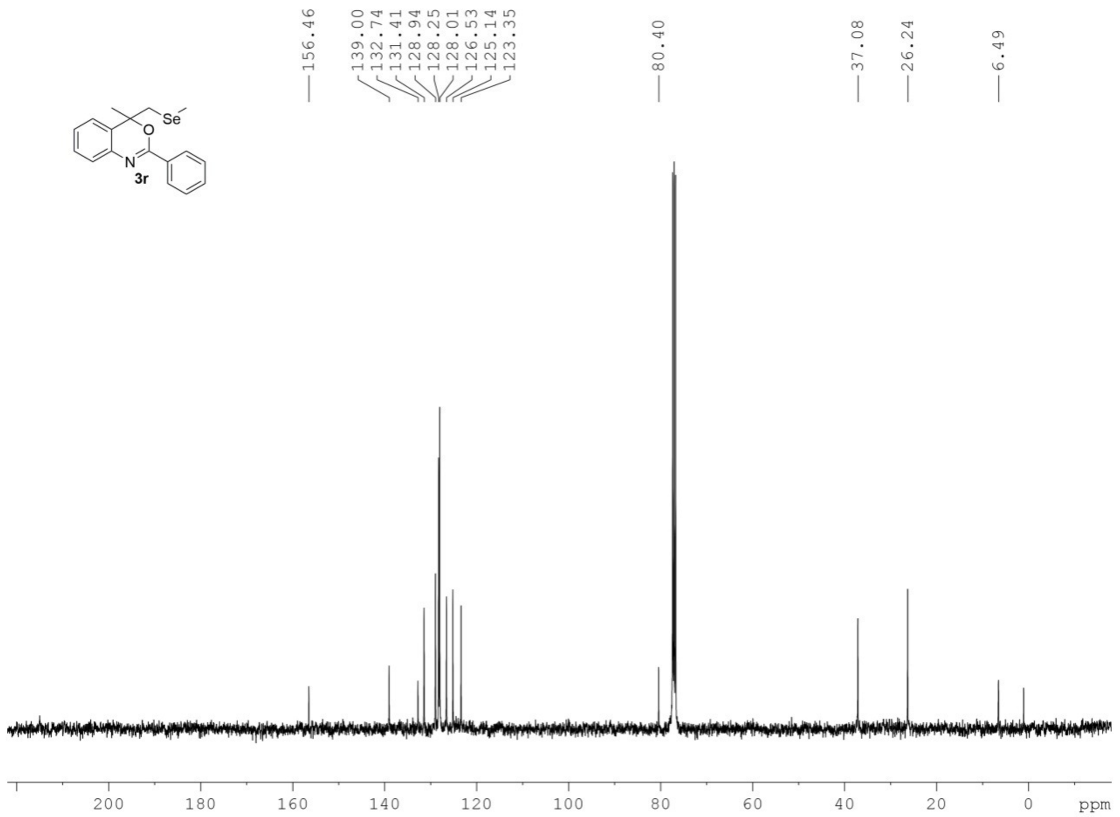

Figure S47. ^{13}C NMR spectrum of **3r**

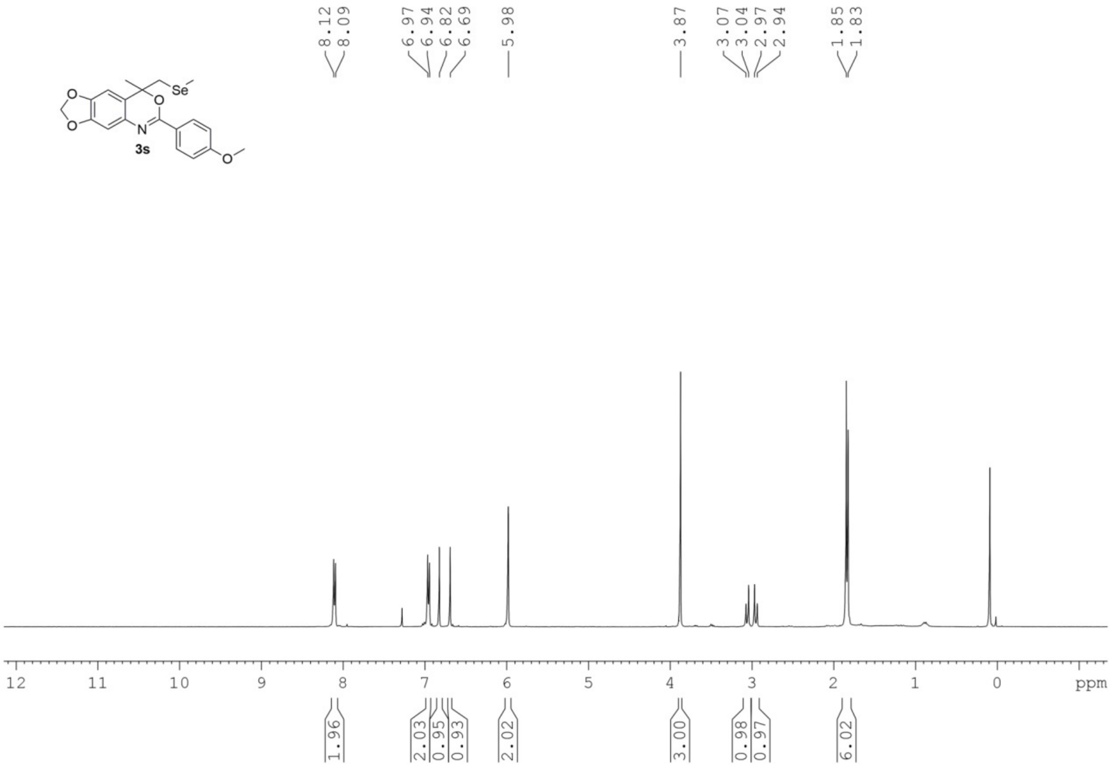

Figure S48. ^{1}H NMR spectrum of **3s**

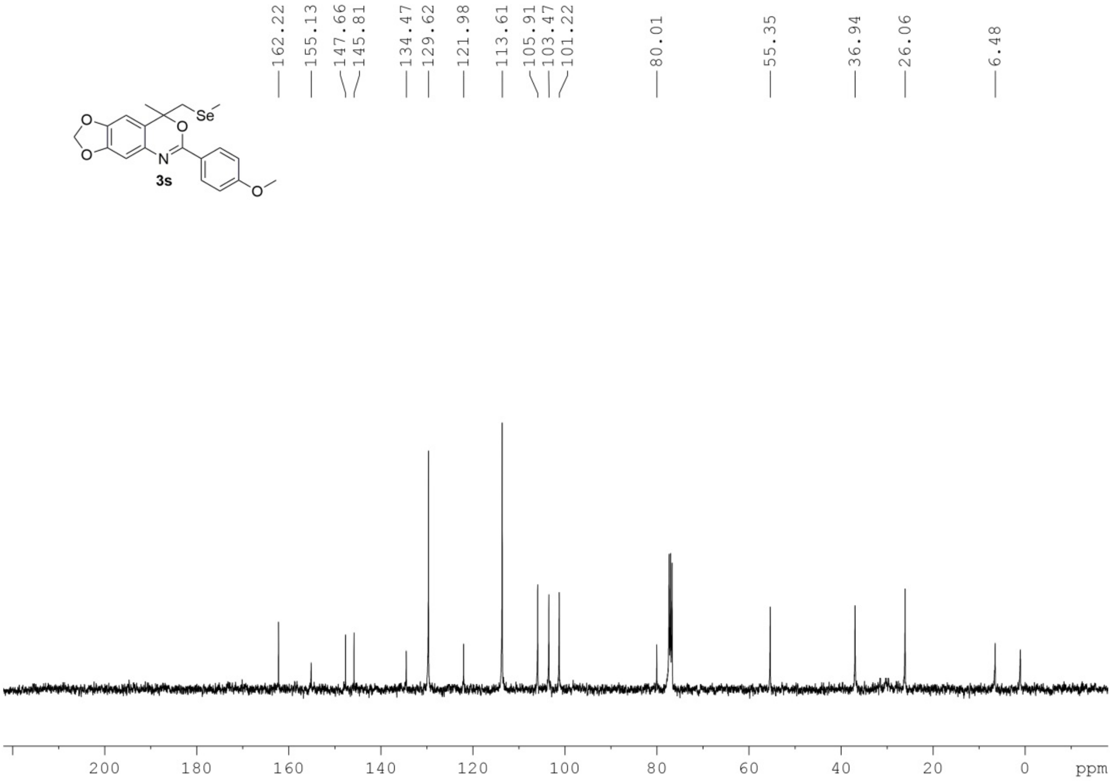

Figure S49. ^{13}C NMR spectrum of **3s**

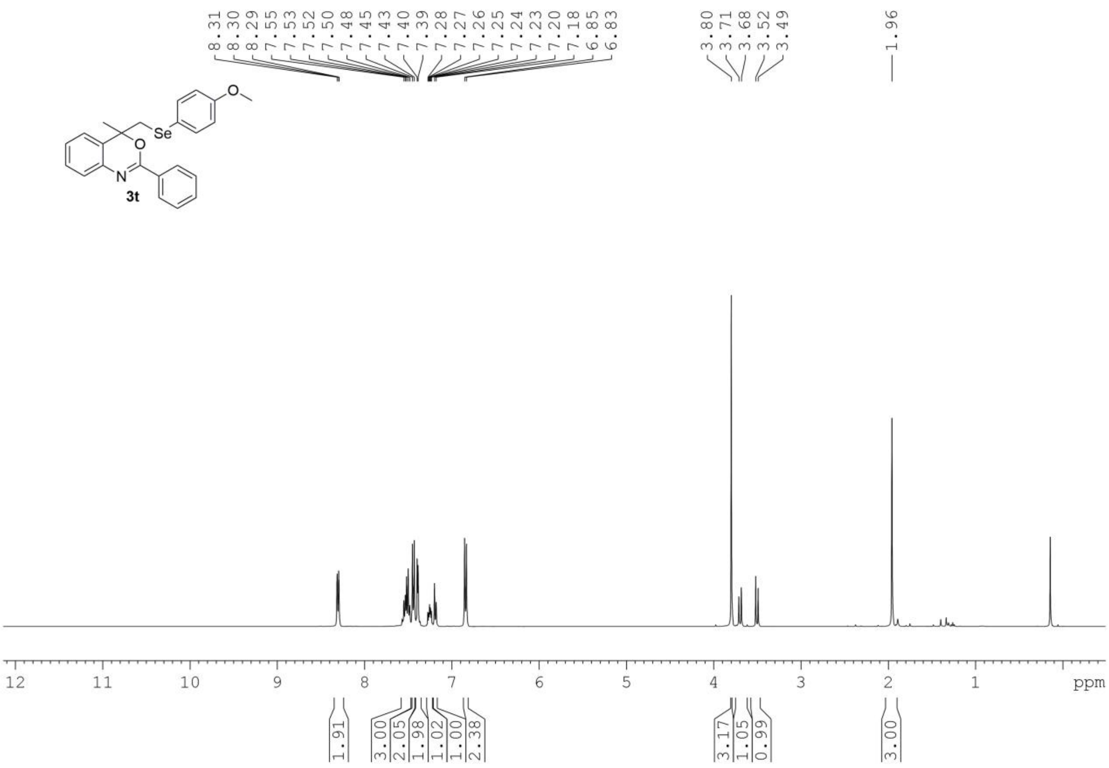

Figure S50. ^{1}H NMR spectrum of **3t**

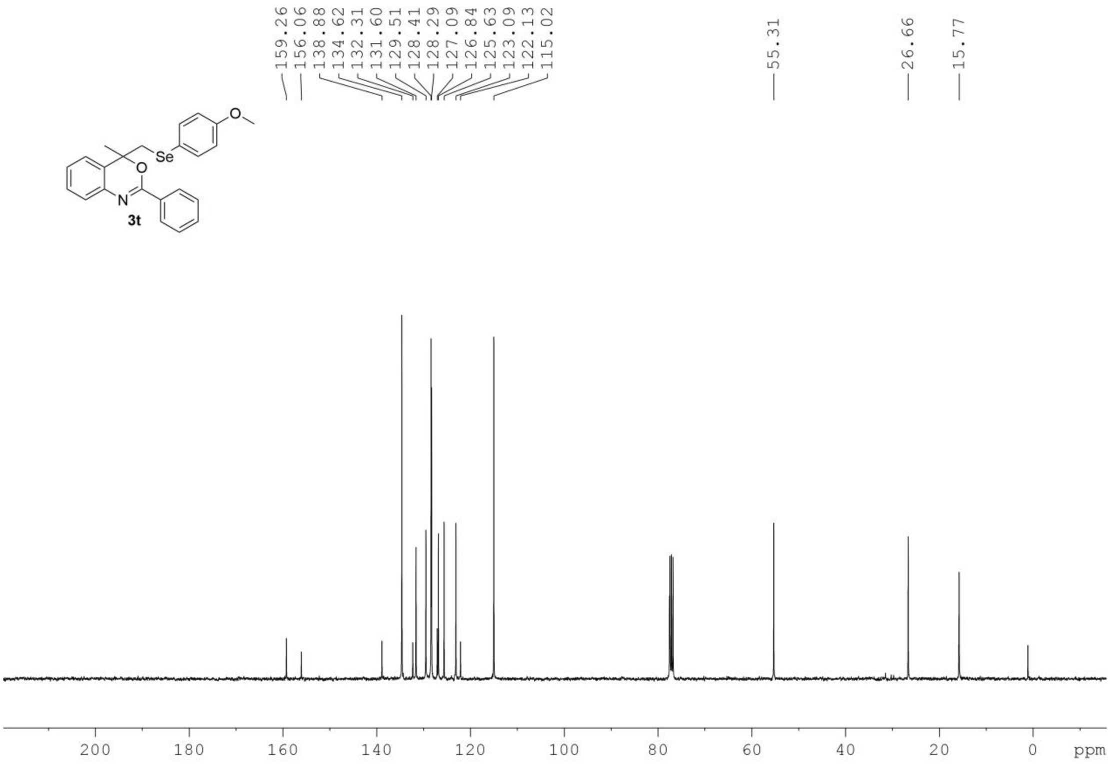

Figure S51. ^{13}C NMR spectrum of **3t**

6. References

1. Guo, J.; Hao, Y.; Li, G.; Wang, Z.; Liu, Y.; Li, Y.; Wang, Q. Efficient synthesis of SCF_3-substituted tryptanthrins by a radical tandem cyclization. *Org. Biomol. Chem.* **2020**, *18*, 1994–2001.
2. Nishio, T. Reaction of (1,ω)-*N*-acylamino alcohols with Lawesson's reagent: Synthesis of sulfur-containing heterocycles. *J. Org. Chem.* **1997,** *62*, 1106–1111.
3. Jana, S; Ashokan, A.; Kumar, S.; Verma, A.; Kumar, S. Copper-catalyzed trifluoromethylation of alkenes: synthesis of trifluoromethylated benzoxazines. *Org. Biomol. Chem.* **2015,** *13*, 8411–8415.
4. Cooper, P.; Crisenza, G. E.; Feron, L.J.; Bower, J.F. Iridium-catalyzed α-selective arylation of styrenes by dual C−H functionalization. *Angew. Chem. Int. Ed.* **2018**, *130*, 14394–14398.
5. Chaitanya, M.; Anbarasan, P. Acid-mediated oxychalcogenation of *o*-vinylanilides with *N*-(arylthio/arylseleno) succinimides. *Org. Lett.* **2018**, *20*, 1183–1186.
6. Okuma, K.; Seto, J.I. Synthesis of indoles, 3,1-benzoxazines, and quinolines from 2-alkenylanilides and active seleniums. *Phosphosus, Sulfur, and Silicon*. **2010**, *185,* 1014–1020.
7. Lu, F.; Xu, J.; Li, H.; Wang, K.; Ouyang, D.; Sun, L.; Huang, M.; Jiang, J.; Hu, J.; Alhumade, H.; Lu, L.; Lei, A. Electrochemical oxidative radical cascade cyclization of olefinic amides and thiophenols towards the synthesis of sulfurated benzoxazines, oxazolines and iminoisobenzofurans. *Green Chem.* **2021**, *23*, 7982–7986.
